# Uncovering the Pre-Deterioration State during Disease Progression Based on Sample-Specific Causality Network Entropy (SCNE)

**DOI:** 10.34133/research.0368

**Published:** 2024-04-29

**Authors:** Jiayuan Zhong, Hui Tang, Ziyi Huang, Hua Chai, Fei Ling, Pei Chen, Rui Liu

**Affiliations:** ^1^ School of Mathematics and Big Data, Foshan University, Foshan 528000, China.; ^2^ School of Biology and Biological Engineering, South China University of Technology, Guangzhou 510640, China.; ^3^ School of Mathematics, South China University of Technology, Guangzhou 510640, China.

## Abstract

Complex diseases do not always follow gradual progressions. Instead, they may experience sudden shifts known as critical states or tipping points, where a marked qualitative change occurs. Detecting such a pivotal transition or pre-deterioration state holds paramount importance due to its association with severe disease deterioration. Nevertheless, the task of pinpointing the pre-deterioration state for complex diseases remains an obstacle, especially in scenarios involving high-dimensional data with limited samples, where conventional statistical methods frequently prove inadequate. In this study, we introduce an innovative quantitative approach termed sample-specific causality network entropy (SCNE), which infers a sample-specific causality network for each individual and effectively quantifies the dynamic alterations in causal relations among molecules, thereby capturing critical points or pre-deterioration states of complex diseases. We substantiated the accuracy and efficacy of our approach via numerical simulations and by examining various real-world datasets, including single-cell data of epithelial cell deterioration (EPCD) in colorectal cancer, influenza infection data, and three different tumor cases from The Cancer Genome Atlas (TCGA) repositories. Compared to other existing six single-sample methods, our proposed approach exhibits superior performance in identifying critical signals or pre-deterioration states. Additionally, the efficacy of computational findings is underscored by analyzing the functionality of signaling biomarkers.

## Introduction

**Fig. 1.**
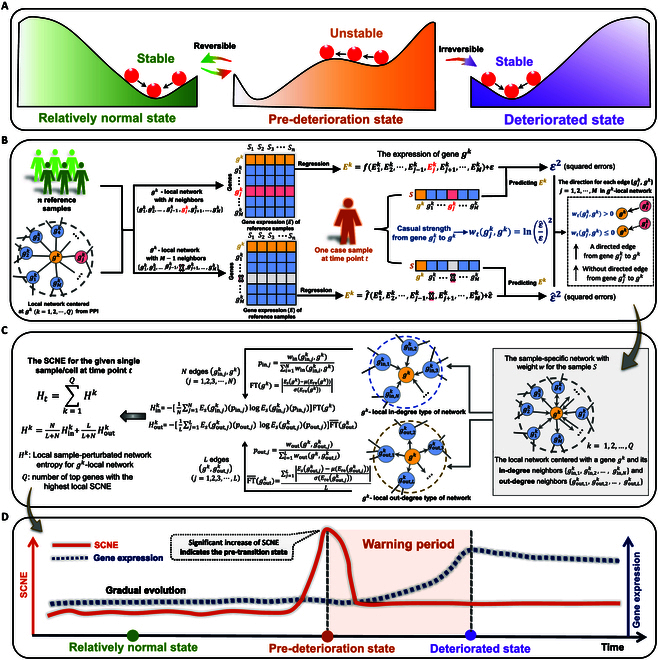
Schematic illustration for uncovering a pre-deterioration state using SCNE. (A) Disease evolution can be broken down into three distinct states: a stable relatively normal state, a pre-deterioration state, and another stable deteriorated state. The pre-deterioration state is distinguished by diminished resilience and heightened susceptibility and is often reversible back to the relatively normal state through suitable intervention. (B) Guided by validation predictions against a group of given reference samples, the construction of the sample-specific causality network is achieved through the utilization of a statistical concept rooted in causal inference. (C) The local SCNE is computed for each localized causality network and provides a quantitative measure of the dynamic changes in causal relations among molecules triggered by a specific sample against the reference samples. (D) The SCNE index is employed to quantify the criticality of complex diseases, and its marked increase serves as an indicator of an approaching pre-deterioration state.

Complex diseases are often the result of alterations in homeostasis induced by environmental or genetic factors. Extensive experimental and clinical evidence indicates that complex disease evolution is not always marked by a gradual pattern but rather distinguished by abrupt and qualitative alterations in the states of the system when reaching a critical transition or tipping point [1,2]. Accordingly, disregarding the particular discrepancies in clinical manifestations and biological mechanisms across diverse ailments, disease evolution can be broken down into three distinct states: a stable relatively normal state, a pre-deterioration state characterized by diminished resilience and heightened susceptibility, and another stable deteriorated state (Fig. 1A). The pre-deterioration state represents the threshold between a relatively normal state and a deteriorated state, underscoring its remarkable importance in disease progression. If complex diseases traverse this pre-deterioration state, a rapid deterioration follows, culminating in the subsequent deteriorated state. Unlike the irreversible deteriorated state, ascertaining the pre-deterioration state can offer the potential to mitigate further deterioration and manage disease progression assistance using well-suited intervention approaches. However, accurately pinpointing the pre-deterioration state or tipping point for complex diseases is a formidable task since there may be negligible alterations in molecular expression or clinical phenotypes prior to disease deterioration [3].

Recently, a quantification method called dynamic network biomarker (DNB) [1,4] has been utilized for the purpose of discerning critical points by harnessing a group of collectively fluctuating molecules during disease progression. The effectiveness of the DNB method is evident in a variety of diseases and biological phenomena, as it has been employed to identify pre-disease states [5,6], detect cell-fate transitions [7,8], and study immune checkpoint blockade [9]. However, the necessity for multiple samples to evaluate statistical conditions presents challenges, as acquiring multi-sample data for each individual in real-world scenarios proves challenging, thus limiting the implementation of DNB approach and its extensions in biological research and clinical contexts. In response to this issue, many different single-sample methods, including single-sample landscape entropy (SLE) [10], single-sample network module biomarkers (sNMB) [11], single-sample-based hidden Markov model (sHMM) [12], temporal network flow entropy (TNFE) [13], personalized dynamic network biomarker (PDNB) [14], and landscape dynamic network biomarker (LDNB) [15], have been developed to quantify the criticality of complex diseases using a specific sample. Nevertheless, those methods mainly focused on identifying early indicators of critical transitions by leveraging the dynamical characteristics of critical states derived from bulk omics data and still encounter challenges related to robustness due to the presence of highly noisy data, especially when working with single-cell data. Hence, there is an urgent demand for the development of the innovative single-sample method that can be suitable for both bulk and single-cell expression data, enabling the detection of pre-deterioration state for complex diseases and the prediction of the key molecules implicated in disease progression.

As high-throughput sequencing technology rapidly advances, many methods, such as cross-map-based framework [16], GRNBoost2 [17], DNRS [18], and NME [19], have been introduced for inferring causal networks. However, these approaches incorporate the inference of causal regulations based on multi-sample data at a specific time, which limits their application to real individualized clinical medicine.

In this study, we propose an innovative quantitative approach called sample-specific causality network entropy (SCNE), which can infer a sample-specific/cell-specific causality network for each sample/cell and effectively function as an indicator of the pre-deterioration state by quantifying the dynamic shifts in causal relations among molecules from the relatively normal state to the critical state. Specifically, guided by validation predictions against a set of reference samples taken from the relatively normal state, the inference of the sample-specific causality network is achieved using a statistical concept rooted in causal inference (Fig. 1B) [20]. Subsequently, the local SCNE is computed for each localized causality network, measuring dynamic changes in causal relations among molecules triggered by a specific sample/cell against the reference samples/cells (Fig. 1C). The criticality of complex disease can be quantified by SCNE, and its marked increase serves as an indicator of an impending critical point or pre-deterioration state (Fig. 1D). To showcase the robustness and efficacy of the SCNE, we conducted a validation of numerical simulations on simulated data subjected to varying levels of noise. As the noise strength increases, our proposed approach exhibits consistent stability and robustness in capturing the imminent critical point when compared to other preexisting single-sample methods [11–15]. Similarly, our SCNE method shows better performance in real-world data, including kidney clear cell carcinoma (KIRC), stomach adenocarcinoma (STAD), and lung adenocarcinoma (LUAD) from the TCGA database. In addition, by applying the SCNE method to the single-cell data of epithelial cell deterioration (EPCD) in colorectal cancer and influenza infection data, we successfully uncovered critical signals of complex diseases, which indicates that the predicted pre-deterioration states manifest before the onset of severe disease deterioration. The above findings align with clinical and experimental observations. Moreover, functional analyses were conducted to assess the validity of the corresponding SCNE signaling biomarkers. In brief, we introduce a new computational method, i.e., SCNE, from the perspective of single-sample data that demonstrates high effectiveness and robustness for the analyses of both bulk and single-cell expression data, providing a fresh perspective for individual disease diagnosis and precision medicine in clinical applications.

## Results

### Validating the effectiveness and robustness of the SCNE method

To assess the effectiveness and resilience of the proposed SCNE approach, an 18-node regulatory network (Fig. S1) controlled by a system of stochastic differential equations shown in Eq. S1 was applied to generate simulated data for the identification of the critical phase as the system is close to a bifurcation point. This regulatory network model, typically expressed in the Michaelis–Menten form, is frequently employed to depict gene regulatory networks across diverse biological phenomena [21,22]. By varying the parameter *s* within the range of −0.50 to 0.15, the simulated data can be generated from the regulatory network. Further details regarding the dynamical system can be found in section A of the Supplementary Materials.

**Fig. 2.**
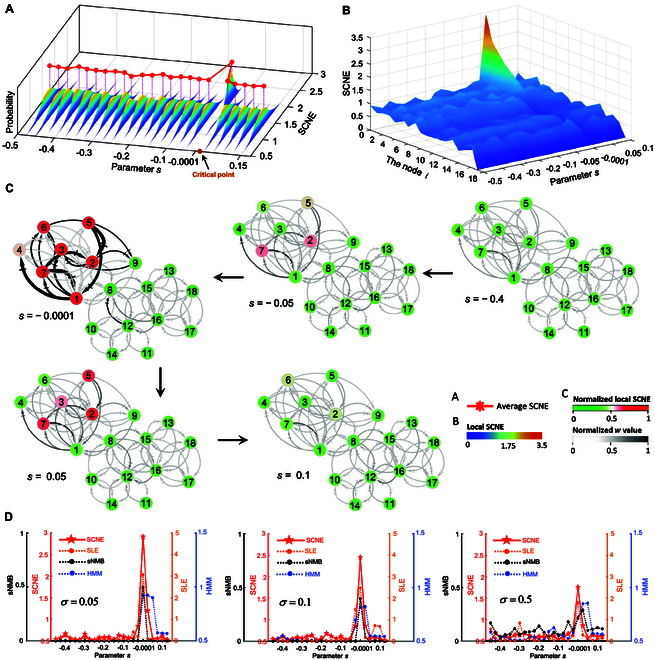
The performance of the SCNE method through numerical simulation. (A) It is evident that the SCNE score suddenly increases near the critical point. (B) The evolution of local SCNE landscapes is depicted for various nodes. Remarkably, the local SCNE of DNB members presents a marked upsurge as the system approaches the bifurcation point. (C) The dynamic evolution of the regulatory network uncovers a notable alteration in the structure of the subnetwork composed of DNB members near the critical point. (D) A comparison between the performance of SCNE and previous single-sample methods is presented. While aiming to detect the critical transition, no obvious distinction is observed between SCNE and the single-sample methods under low noise levels (*σ* = 0.05 or 0.1). However, when subjected to high noise levels (*σ* = 0.5), the SCNE method demonstrates heightened robustness and efficacy.

As depicted in Fig. 2A, an abrupt and steep surge in the SCNE score was observed near the specific parameter *s* = 0 (the bifurcation point), signifying the indicator of an impending critical point. To enhance the visual representation of the distinct dynamics between the relatively normal and pre-deterioration states, the landscape evolution of local SCNE for various nodes is presented (Fig. 2B). Evidently, as the system is distant from the critical point, the local SCNE scores of all nodes remain consistently low, but there is a sharp increase in the local SCNE of specific nodes (namely, DNB members) when the system approaches the critical point. Moreover, Fig. 2C shows the dynamic evolution of the regulatory network, where a distinct alteration in the configuration of the subnetwork consisting of DNB members occurs near the critical point, indicating an imminent shift in the state of the network. Additionally, to showcase the resilience of the proposed method, we conducted a comparative analysis between SCNE and other existing single-sample methods [11–13] using samples perturbed with varying levels of noise (Fig. 2D). As the noise strength increased, the SCNE method exhibited enhanced robustness and effectiveness in the identification of the critical point in biological processes, as evidenced by its consistent ability to yield critical signals with higher sensitivity and apparent scores in the presence of stronger noise. Additionally, a six-node regulatory network was used to generate simulated data to explore the relationship between the pivotal indices of the DNB theory and those of our SCNE index (Figs. S3 and S4). The above numerical simulations illustrate that our proposed SCNE method has the capability to extract high-dimensional information solely from a specific sample.

### Identifying pre-deterioration states of individual influenza infection

**Fig. 3.**
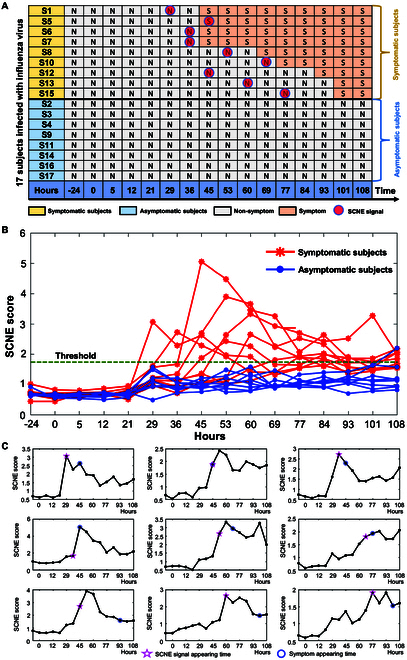
Identification of the state for influenza infection individuals. (A) Summary table presenting comprehensive information for the cohort of 17 individuals within the influenza infection dataset. (B) Curve of SCNE scores for 17 individuals. The red curve signifies SCNE scores for the nine symptomatic subjects, whereas the blue curve corresponds to SCNE scores for the eight asymptomatic subjects. (C) Individualized SCNE score curves for nine symptomatic individuals. The blue circles mark the time of initial flu symptoms as observed clinically, while the orange stars denote the pre-deterioration state identified by SCNE analysis.

In the time-course dataset related to influenza infection, a group of 17 individuals was categorized into two distinct classes: 9 symptomatic subjects developing clinical symptoms of influenza infection and 8 asymptomatic subjects showing no clinical symptoms. The gene expression data for these individuals were acquired at 16 distinct sampling time points spanning from −24 to 108 h, as shown in Fig. 3A. In the case of each individual, the gene expression profiles from the preceding four time points were treated as a reference group, representing their comparatively healthy condition. The individualized SCNE score (defined as *H_t_* in Eq. 9) was calculated for each of the 17 individuals by applying the algorithm described in Materials and Methods. The increase in the SCNE score serves as an early warning sign for disease onset, specifically indicating the stage at which clinical symptoms appear. For the group of nine symptomatic subjects, there was a notable increase in the SCNE score before the onset of influenza symptoms, whereas the score remained comparatively steady for the eight asymptomatic subjects (Fig. 3B). Consequently, the emergence of early warning signals for influenza symptoms was observable in the group of nine symptomatic subjects, while no such signals were discernible in the eight asymptomatic subjects. Figure 3C presents the individualized SCNE scores of the nine symptomatic subjects, uncovering the pre-deterioration state prior to the onset of clinical symptoms in each symptomatic individual. Hence, our SCNE approach convincingly demonstrates its capability to effectively and accurately identify pre-deterioration states for individual influenza virus infections.

### Identifying pre-deterioration states of tumor diseases

**Fig. 4.**
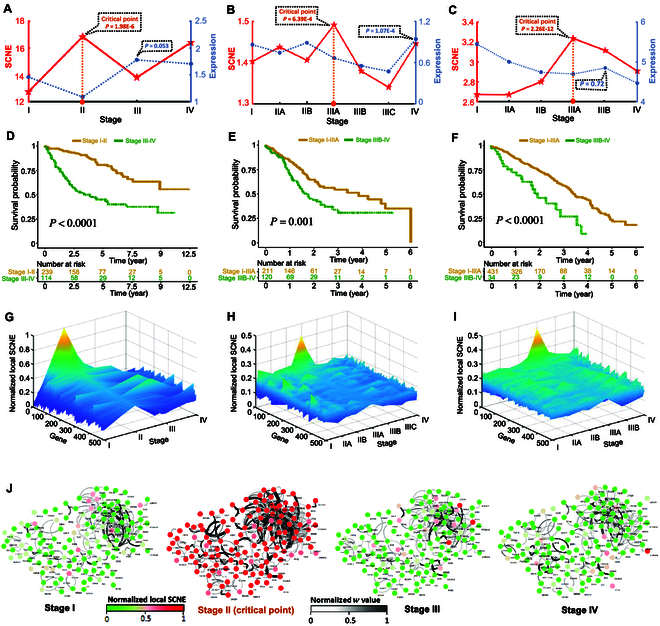
Identification of the pre-deterioration state for tumor diseases. The dynamic performance of SCNE and gene expression were analyzed in three tumor types: (A) KIRC, (B) STAD, and (C) LUAD. Survival time before and after the pre-deterioration states are compared in three tumor types: (D) KIRC, (E) STAD, and (F) LUAD. Landscapes depicting the local SCNE score for both signaling and nonsignaling genes were generated for three datasets: (G) KIRC, (H) STAD, and (I) LUAD. (J) Dynamic evolution of signaling gene networks is presented in the KIRC dataset.

To assess the effectiveness of the SCNE method in identifying pre-deterioration states of tumor diseases, we employed this approach for three tumor datasets (KIRC, STAD, and LUAD) obtained from The Cancer Genome Atlas (TCGA) database. For each individual tumor sample, we calculated the individual-specific SCNE score (as defined in Eq. 9) using the algorithm described in Materials and Methods. The average SCNE value at each stage was used as a quantitative measure of the pre-deterioration state. Our proposed method analysis revealed that the pre-deterioration state was determined to be stage II for KIRC, stage IIIA for STAD, and stage IIIA for LUAD (Fig. 4A to C). Specifically, for the KIRC dataset, as shown in Fig. 4A, a remarkable increase (*P* =1.98E − 6) in the SCNE score was evident between stages I and II, implying the occurrence of a critical deterioration event after stage II. Indeed, stage III is characterized by a swift escalation in the lipid levels surrounding the kidney, followed by the tumor invading the renal vein [23]. In the STAD dataset, a significant transition (*P* = 6.39E − 4) in the SCNE score occurred at stage IIIA (Fig. 4B), after which the tumor infiltrated adjacent tissues or spread to distant organs, eventually leading to distant metastasis [24]. Similarly, in the LUAD dataset, a sharp increase (*P* = 2.26E − 12) in the SCNE score at stage IIIA indicates an upcoming critical deterioration, which aligns with the observation that tumor cells exhibit the capability to infiltrate tissues or organs distant from the primary site during stages IIIB to IV [25]. However, as observed in the curve represented by the dark blue color in Fig. 4A to C, the gene expression of differentially expressed genes does not demonstrate a signal of critical transition. Moreover, our proposed approach exhibits better performance in uncovering pre-deterioration states during disease progression in comparison to other existing six single-sample methods [11–15] (Table and Fig. S6). In addition, a comparative analysis was conducted between our proposed SCNE and another method that utilizes the background protein–protein interaction (PPI) network without the introduced distinctions. The result indicates that the signal provided by our proposed SCNE method is significantly stronger than that from the alternative method (Fig. S7).

**Table.** Comparison of the performance among different single-sample detection methods

Dataset	KIRC	STAD	LUAD	EPCD
SCNE	Stage II(*P* = 1.98E−6)	Stage IIIA(*P* = 6.39E−4)	Stage IIIA(*P* = 2.26E−12)	Cluster 4(*P* = 1.43E−10)
SLE	None	Stage IIIA(*P* = 0.039)	Stage IIIB(*P* = 5.62E−6)	Cluster 6(*P* = 2.18E−5)
sNMB	None	Stage IIIA(*P* = 0.038)	Stage IIIB(*P* = 2.64E−4)	Cluster 4(*P* = 2.47E−5)
sHMM	Stage II(*P* = 1.04E−5)	Stage IIIA(*P* = 0.046)	Stage IV(*P* = 3.39E−5)	None
TNFE	Stage IV(*P* = 1.35E−66)	Stage IIIC(*P* = 0.016)	Stage IIIB(*P* = 0.0012)	None
PDNB	None	Stage IIIA(*P* = 0.034)	Stage IV(*P* = 0.021)	Cluster 5(*P* = 0.0056)
LDNB	Stage II(*P* = 0.016)	Stage IV(*P* = 0.033)	Stage IIIB(*P* = 3.42E−04)	Cluster 6(*P* = 0.024)

To validate the identified pre-deterioration state, we performed prognostic analysis for samples separately derived from before and after the critical transition based on Kaplan–Meier (log-rank) survival analysis. As depicted in Fig. 4D to F, the survival curves before and after the pre-deterioration state exhibited noticeable differences, with significant *P* values observed for KIRC, STAD, and LUAD (*P* < 0.0001, *P* = 0.001, and *P* < 0.0001, respectively). These results highlight that patients diagnosed before the identified pre-deterioration state demonstrate significantly improved prognoses compared to those diagnosed after reaching the pre-deterioration stage. More detailed information regarding the survival analysis of tumors can be found in Figs. S8 and S9. Thus, the SCNE score has the ability to identify early warning signals for pre-deterioration states associated with survival time. Furthermore, to provide a comprehensive overview of the alterations in the local SCNE, the landscape of local SCNE for both signaling and nonsignaling genes is displayed in Fig. 4G to I, where a cluster of signaling genes demonstrates a sudden surge in local SCNE at pre-deterioration states or critical points. In addition, as depicted in Fig. 4J, we illustrate a visual representation of the dynamic evolution of the network constructed with signaling genes (the top 5% genes with the largest SCNE score) for the KIRC dataset. A noticeable shift in the network structure occurs at the critical point, signifying the critical transition toward disease deterioration.

### Identifying pre-deterioration states of EPCD in colorectal cancer

**Fig. 5.**
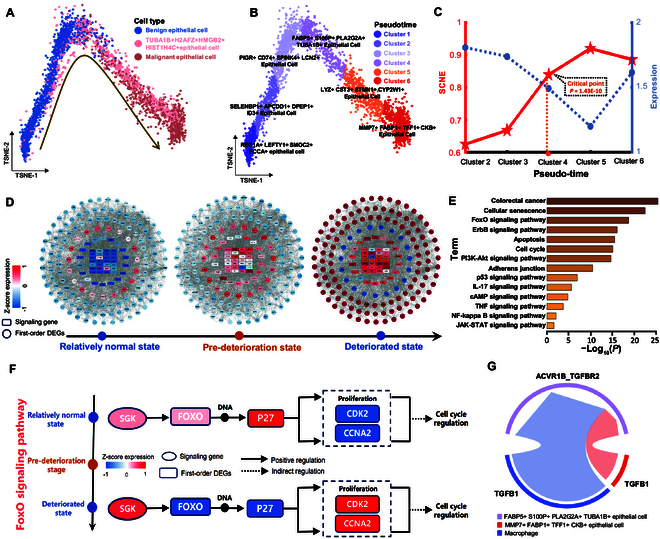
Identification of the pre-deterioration state for EPCD in colorectal cancer. (A) A deterioration trajectory for epithelial cells was constructed based on three distinct subpopulations. (B) The progression of EPCD was categorized into six different periods or clusters. (C) The dynamic performance of SCNE and gene expression were analyzed in the development of EPCD. (D) The dynamic evolution of the regulatory network formed by signaling genes and their neighboring DEGs was investigated during the process of EPCD. (E) Enrichment analysis of KEGG pathways was conducted for the first-order differentially expressed neighbors. (F) The functional analysis performed on the genes and their differentially expressed neighbors uncovered the signaling mechanism linked to cell cycle regulation in the FoxO signaling pathway. (G) Cellular communication among distinct cell subpopulations via the TGF-β signaling pathway.

To gain deeper insight into the molecular mechanisms underlying colorectal carcinogenesis, a trajectory of EPCD was established using three distinct subpopulations: benign cells, *TUBA1B*+*H2AFZ*+ *HMGB2*+ *HIST1H4C*+ cells, and malignant cells (Fig. 5A). By analyzing patterns of gene expression, the progression of EPCD was categorized into six different periods or clusters (Fig. 5B). Specific details have been provided in our previous study [26]. Figure 5C indicates that a notable shift (*P* = 1.43E − 10) in the SCNE score occurs in cluster 4, which indicates a critical signal of transition into EPCD and reveals a subpopulation of pre-deteriorated epithelial cells. Furthermore, our proposed SCNE demonstrates superior performance in detecting pre-deterioration states during EPCD when compared to the other existing six single-sample methods (Table). In the identified pre-deterioration state, a subset of genes representing the top 5% with the most elevated local SCNE values were chosen as signaling genes. Furthermore, the regulatory network constructed by signaling genes and their neighboring differentially expressed genes was employed to explore the network-level molecular regulatory mechanism underlying tumor progression. As depicted in Fig. 5D, a noticeable shift in gene expression patterns within the networks becomes apparent after the pre-deterioration state, where gene expression levels demonstrate distinct changes, transitioning from either high to low or the reverse. Figure 5E reveals that these neighboring differentially expressed genes (DEGs) exhibited significant enrichment in cancer-related signaling pathways, including the phosphatidylinositol 3-kinase (PI3K)–Akt signaling pathway [27], cellular senescence [28], and the FoxO signaling pathway [29].

Figure 5F shows that the functional analysis conducted for the core genes and their first-order neighbors reveals the underlying mechanism associated with cell cycle regulation in the FoxO signaling pathway. The FoxO signaling pathway represents an equilibrium-driven mechanism responsible for governing cell proliferation and apoptosis and has a close interrelation with the development, invasion, and metastasis of diverse tumor types [30,31]. As depicted in Fig. 5F, during the deterioration process of epithelial cells, an evident increase in the expression of the signaling gene *SGK* was observed, while the expression of the first-order neighbors *FOXO* and *P27* was markedly reduced, indicating a negative regulatory role of *SGK* on *FOXO*. Subsequently, the expression levels of downstream molecules, including proliferation-related genes such as *CDK2* and *CCNA2*, were a markedly increased, which implies the potential disruption of the cell cycle and the propensity for aberrant proliferation during the progression of EPCD. Therefore, activated *SGK* primarily triggers conformational changes in the *FOXO* transcription factor through phosphorylation, resulting in the attenuation of its transcriptional activity. This intricate process leads to a reduction in the expression of the cell cycle inhibitory factor *P27*, subsequently fostering abnormal cell proliferation and bolstering the survival and propagation of cancer cells [32]. It has been demonstrated that the expression level of *P27* is substantially higher in benign lesions and normal tissues than in malignant tissues [33], which is in accordance with our research results. In summary, the SGK gene exerts inhibitory effects on the expression of the first-order neighboring *FOXO*, ultimately leading to the down-regulation of the cell cycle inhibitory factor *P27*. This molecular cascade ultimately culminates in the manifestation of aberrant cell proliferation, potentially contributing to the progression of precursor epithelial cells from a state of deterioration to terminal malignant epithelial cells. As depicted in Fig. 5G, in the context of the transforming growth factor-β (TGF-β) signaling pathway, the *MMP7*+ *FABP1*+ *TFF1*+ *CKB*+ epithelial cell subset (cluster 6) and the macrophage subset communicate a robust proliferation signal to the *FABP5*+ *S100P*+ *PLA2G2A*+ *TUBA1B*+ epithelial cell subset (cluster 4), potentially indicating an impending exacerbation in the deterioration of epithelial cells. Moreover, within the tumor microenvironment (TME), macrophages exhibit the highest receptor–ligand communication probability in the TGF-β signaling pathway (Fig. S1o), implying their potential contribution to the propagation of EPCD. Additionally, TGF-β can modulate the activity of the FoxO signaling pathway by regulating interactions between *FOXO* and other proteins, thereby influencing cell proliferation and survival [34]. *TGF-β1* can form a stable heterotetrameric complex with *TGFBR2* and *TGFBR1*, which plays a crucial role in modulating the proliferation dynamics of epithelial cells [35]. In our research, *TGF-β1* acted as a signaling gene, while *TGFBR2* and *TGFBR1* served as the first-order neighbors. Their reciprocal interactions and potential feedback to other signaling genes may ultimately contribute to the modulation of intracellular communications and the dynamics of cell proliferation.

## Discussion

The detection of critical signals for abrupt deterioration is pivotal in the majority of complex diseases. However, the application of existing critical-state detection methods to bulk RNA-sequencing data with limited sample sizes or single-cell data is constrained by the substantial noise inherent in the data [36]. In this research, we present a robust computational method at the specific sample level, called SCNE, that is capable of constructing a sample-specific causality network for each individual and efficiently identifying critical points or pre-deterioration states associated with disease deterioration. By implementing the SCNE method for simulated datasets and five real datasets, we effectively pinpointed the critical signals of imminent severe deterioration. The successful predictions in these datasets validate the effectiveness of the SCNE score in quantifying critical points solely based on specific samples. In addition, the robustness of SCNE was validated via numerical simulation under different noise strengths, and its good performance in detecting disease-related critical points was demonstrated through comparisons with existing single-sample methods using tumor datasets.

The benefits of our SCNE method can be succinctly outlined as follows. First, by introducing SCNE, our approach effectively mitigates the impact of substantial noise present in omics data, consequently bolstering its robustness and reliability. Second, the proposed approach infers a sample-specific causality network and provides its SCNE score to quantify the criticality of complex diseases, which has better performance in uncovering the pre-deterioration state during disease progression compared to other existing six single-sample methods. Third, the SCNE approach not only serves as an indicator for critical shifts toward a deteriorated state but also pinpoints corresponding signaling biomarkers implicated in vital biological processes. Finally, as a model-free computational approach, SCNE is versatile in its applicability to various omics data types, including both bulk and single-cell datasets. However, limitations of the SCNE method include its dependency on a PPI network as the background network and the necessity for a reference group composed of relatively healthy samples (see section B of Supplementary Materials for details). In addition, the precise interpretation of the biomedical significance of each critical point in a complex multi-stage disease process remains an unresolved aspect and will be a focus of our forthcoming research endeavors.

## Materials and Methods

### Theoretical background

From the perspective of the dynamical system, disease evolution can generally be understood as a time-evolving nonlinear dynamical process, during which a suddenly deteriorating state is seen as a qualitative change in state taking place at a bifurcation point [37]. Thus, such a development process is typically broken down into three states (Fig. 1A): (a) a stable relatively normal state prior to the critical transition; (b) a pre-deterioration state with heightened sensitivity to disruptions, signifying a critical transition of severe disease deterioration; and (c) another stable deteriorated state marked by disease onset or deterioration. Generally, there is no noticeable distinction between the relatively normal state and pre-deterioration state, in contrast to the deteriorated state. Consequently, conventional statistical approaches such as differential gene expression analysis might face challenges in distinguishing the pre-deterioration state (Fig. 1D).

Recently, the theoretical concept of DNB [1,4] has emerged and was developed to quantitatively identify the critical state or tipping point during the progression of a complex system based on multi-sample data. Particularly, when a complex system is near the critical point, DNB molecules mainly satisfy the following two statistical conditions [38]: The standard deviation for DNB molecule drastically increases, and the correlation between DNB molecules rapidly increases. Actually, the properties of DNB suggest that the critical transition of a system can be signaled by fluctuations in molecule expression and alterations in their causal regulatory strength [39]. Therefore, we analyzed the constructed causal networks in our study to demonstrate dynamic changes in both the expression fluctuation and causal strength, signaling an impending deterioration of complex diseases. In our study, when the system approaches critical state, there exists a group of dominant variables defined as the DNB molecules, which satisfy the following two criteria based on the observed data: (a) The expression deviation or fluctuation of dominant variables drastically increases, and (b) the causal strength between dominant variables rapidly increases (see section A of the Supplementary Materials for details).

From the perspective of a sample-specific causality network, our proposed SCNE method was intentionally developed to pinpoint critical signals delineating the shift from the relatively normal state to the deteriorated state. Based on a statistical concept for causal inference [20], SCNE can enable the inference of causal effects among molecules through validation prediction analysis and reconstruct the sample-specific causality network. Specifically, our proposed method utilizes the PPI network as a background, integrating individualized gene expression data to infer the sample-specific causality network. Notably, dynamic regulatory relationships between genes are taken into account during the construction of the sample-specific causality network. We have carried out an analytical demonstration to explore how the network topology and dynamics change leading up to the pre-deterioration state, identifying key dynamic molecules that play a crucial role in driving the system toward deterioration (shown in Fig. S5 and Tables S1 and S2). Overall, the SCNE approach has the capability to serve as a strong indicator of the pre-deterioration state by quantifying the dynamic changes of the constructed causal network. The source code of the algorithm is freely available at https://github.com/zhongjiayuan/SCNE_project.

### Algorithm for uncovering the pre-deterioration state using SCNE

With a collection of reference samples depicting a comparatively healthy stage, the computational approach SCNE was implemented to uncover the critical point or pre-deterioration state using a case sample, and a comprehensive description of its process is elucidated in the subsequent sections.

[Step 1] Construction of the sample-specific causality network *N_S_* for the case sample at time point *t*. By utilizing the PPI network and given reference samples, the construction of a sample-specific causality network for the case sample can be achieved by a causal strength index $w_{t}\left(g_{j}^{k},g^{k}\right)$, as defined in Eq. 1. Specifically, if the $w_{t}\left(g_{j}^{k},g^{k}\right)$ value exceeds zero, it indicates the existence of a directed edge $\left(g_{j}^{k},g^{k}\right)$ from gene $g_{j}^{k}$ to *g^k^*; otherwise, there is the absence of such a directed edge. Thus, by determining the direction of each edge $\left(g_{j}^{k},g^{k}\right)$ based on the causal strength index $w_{t}\left(g_{j}^{k},g^{k}\right)$, we construct a sample-specific causality network *N_S_* for a case sample at time point *t*.

$$w_{t}\left(g_{j}^{k},g^{k}\right)=\ln{\left(\frac{\hat{\varepsilon }}{\varepsilon }\right)}^{2}\;$$ (1)

where $\;\hat{\varepsilon }\;$ and *ε* denote the test errors obtained from Eqs. 2 and 3, respectively, when applied to the case sample.

$$\begin{array}{c} E^{k}=\hat{f}\left(Z^{k}\right)+\hat{\varepsilon }=a_{1}E_{1}^{k}+a_{2}E_{2}^{k}+\cdots \\ \;+a_{j-1}E_{j-1}^{k}+a_{j+1}E_{j+1}^{k}+\cdots +a_{M}E_{M}^{k}+\hat{\varepsilon }, \end{array}$$ (2)

$$\begin{array}{c} E^{k}=f\left(E_{j}^{k},Z^{k}\right)+\varepsilon =b_{1}E_{1}^{k}+b_{2}E_{2}^{k}+\cdots \\ \;+b_{j-1}E_{j-1}^{k}+b_{j}E_{j}^{k}+b_{j+1}E_{j+1}^{k}+\cdots +b_{M}E_{M}^{k}+\varepsilon , \end{array}$$ (3)

where symbol $E_{j}^{k}$ represents the expression of the *j*th gene $g_{j}^{k}$ of the local network *N^k^* centered with the *k*th gene *g^k^*, and vector ***Z***^***k***^ is defined as $\left(E_{1}^{k},E_{2}^{k},\;\cdots ,E_{j-1}^{k},E_{j+1}^{k},\cdots ,E_{M}^{k}\right)$ with *a_i_* (*i* = 1, 2, ⋯, *j* − 1, *j* + 1, ⋯, *M*) representing regression coefficients of $\hat{f}$. Similarly, *b_i_* (*i* = 1, 2, ⋯, *M*) denotes regression coefficients of *f*. Specifically, for a local network *N^k^* centred with a gene *g^k^* in the PPI network, whose first-order neighbors are genes $\left\{g_{1}^{k},\;g_{2}^{k},\;\cdots ,\;g_{j-1}^{k},g_{j}^{k},g_{j+1}^{k},\cdots ,g_{M}^{k}\right\}$, we assume that all the first-order neighbors $g_{j}^{k}$ (*j* = 1, 2, ⋯, *M*) are the cause of the center node *g^k^*, i.e., the change of expressions of any neighbor $g_{j}^{k}$ may affect that of *g^k^*. A group of relative healthy reference samples serves as the training samples to determine regression models $\hat{f}$ and *f*, while a single case sample at each time point *t* is designated as the test sample. By inputting the test sample into $\hat{f}$ and *f*, respectively, the output $\;\hat{\varepsilon }=E^{k}-\hat{f}\left(Z^{k}\right)$ and $\varepsilon =E^{k}-f(\;E_{j}^{k},Z^{k}\;)$ are obtained to infer the sample-specific causality network. A more detailed description is given in section E of the Supplementary Materials.

[Step 2] Extraction of each local causality network from the sample-specific causality network *N_S_*. The local causality network is composed of two types of networks: the local in-degree network and the local out-degree network. Specifically, the *g^k^*-local causality network ${\mathrm{LN}}_{S}^{k}\;$ is centered at the gene *g^k^*, which has *N* first-order in-degree neighbors $\left\{g_{\text{in},1}^{k},g_{\text{in},2}^{k},\ldots ,g_{\text{in},N}^{k}\right\}$ corresponding to *N* in-degree edges and *L* first-order out-degree neighbors $\left\{g_{\text{out},1}^{k},g_{\text{out},2}^{k},\ldots ,g_{\text{out},L}^{k}\right\}$ corresponding to *L* out-degree edges. The edge weights between the central gene *g^k^* and its first-order in-degree neighbors, denoted as $\left\{w_{\text{in}}\left(g_{\text{in},1}^{k},g^{k}\right),\;w_{\text{in}}\left(g_{\text{in},2}^{k},g^{k}\right),\;\ldots ,\;w_{\text{in}}\left(g_{\text{in},N}^{k},g^{k}\right)\right\}$, as well as its first-order out-degree neighborhood genes, denoted as $\;\left\{w_{\text{out}}\left(g^{k},g_{\text{out},1}^{k}\right),\;w_{\text{out}}\left(g^{k},g_{\text{out},2}^{k}\right),\;\ldots ,\;w_{\text{out}}\left(g^{k},g_{\text{out},L}^{k}\right)\right\}$, are determined by the causal strength index *w_t_*.

[Step 3] Calculation of a local SCNE score for each local causality network. Specifically, in the context of the *g^k^*-local causality network ${\mathrm{LN}}_{S}^{k}$(comprising *N* first-order in-degree neighbors and *L* first-order out-degree neighbors), its local SCNE score is computed through the following equation (Eq. 4).

$$H^{k}=\frac{N}{L+N}H_{\text{in}}^{k}+\frac{L}{L+N}H_{\text{out}}^{k},$$ (4)

where the definitions for $H_{\text{in}}^{k}$ and $H_{\text{out}}^{k}$ are provided below.

$$\begin{array}{c} H_{\text{in}}^{k}=\\ -\left[\;\frac{1}{N}\sum_{j=1}^{N}E_{s}\left(g_{\text{in},j}^{k}\right)\left(p_{\text{in},j}\right)\text{log}E_{s}\left(g_{\text{in},j}^{k}\right)\left(p_{\text{in},j}\right)\right]\text{FT}\left(g^{k}\right) \end{array}$$ (5)

with

$$p_{\text{in},j}=\frac{w_{\text{in}}\left(g_{\text{in},j}^{k},g^{k}\right)}{\sum_{i=1}^{N}w_{\text{in}}\left(g_{\text{in},i}^{k},g^{k}\right)},\text{FT}\left(g^{k}\right)=\left|\frac{E_{s}\left(g^{k}\right)-\mu \left(E_{\mathrm{re}}\left(g^{k}\right)\right)}{\sigma \left(E_{\mathrm{re}}\left(g^{k}\right)\right)}\right|\;$$ (6)

and

$$\begin{array}{c} H_{\text{out}}^{k}=\\ -\left[\;\frac{1}{L}\sum_{j=1}^{L}E_{s}\left(g_{\text{out},j}^{k}\right)\left(p_{\text{out},j}\right)\text{log}E_{s}\left(g_{\text{out},j}^{k}\right)\left(p_{\text{out},j}\right)\right]\bar{\text{FT}}\left(g_{\text{out}}^{k}\right) \end{array}$$ (7)

with

$$\begin{array}{c} p_{\text{out},j}=\frac{w_{\text{out}}\left(g^{k},g_{\text{out},j}^{k}\right)}{\sum_{i=1}^{L}w_{\text{out}}\left(g^{k},g_{\text{out},i}^{k}\right)},\;\\ \bar{\text{FT}}\left(g_{\text{out}}^{k}\right)=\frac{\sum_{j=1}^{L}\left|\frac{E_{s}\left(g_{\text{out},j}^{k}\right)-\mu \left(E_{\mathrm{re}}\left(g_{\text{out},j}^{k}\right)\right)}{\sigma \left(E_{\mathrm{re}}\left(g_{\text{out},j}^{k}\right)\right)}\right|}{L}\;, \end{array}$$ (8)

where *E_s_*(*g^k^*) represents the gene expression of the central gene *g^k^* in the case sample, while *μ*(*E*_re_(*g^k^*)) and *σ*(*E*_re_(*g^k^*)) correspond to the mean and variance of gene expression for the central gene *g^k^* in the reference samples, respectively. Similarly, $E_{s}\left(g_{\text{out},j}^{k}\right)$ represents the gene expression of the *j*th out-degree neighbor $g_{\text{out},j}^{k}\;$ of ${\mathrm{LN}}_{S}^{k}$ in the case sample *S*. FT(*g^k^*) can be seen as quantifying the expression fluctuation/deviation of the gene *g^k^* in the case sample against the reference samples (see section F of the Supplementary Materials for details).

[Step 4] Calculation of an SCNE score for the case sample at a specific time point *t*. To be more precise, when considering a subset of genes with the most elevated local SCNE score, the SCNE score for the particular sample can be derived using the following formula:

$$H_{t}=\sum_{k=1}^{Q}H^{k}$$ (9)

where constant *Q* represents a configurable parameter set to the number of the top 5% genes with the highest local SCNE scores. When the system approaches the critical state, there is a noticeable change in the network structure of the subnetwork composed of specific variables (DNB members), characterized by a marked increase in expression fluctuation (FT) of DNB molecules and a rapid rise in causal strength (*w* value) among them (Fig. S5). By exploring the dynamic information of such a group of DNB variables at a network level, it becomes possible to predict the qualitative state transition. Thus, the *H_t_* index is designed to quantify the expression fluctuation and causal strength variation triggered by each single sample against a group of given reference samples, providing the warning signals of the pre-deterioration state.

[Step 5] Identification of the pre-deterioration state using the one-sample *t* test. To evaluate the capacity of the SCNE score in capturing critical dynamics, we employ the one-sample *t* test [40] to ascertain whether a statistically significant distinction exists between the relatively normal and pre-deterioration states. Specifically, the following one-sample *t* test statistic *S* is used to be a statistical indicator of distinction between a value *z* and the mean of an *n*-dimensional vector $\hat{Z}=(z_{1},\;z_{2},\;\cdots ,z_{n})$.

$$S=\frac{\left(\text{mean}\left(\hat{Z}\right)-z\right)\sqrt{n}}{\mathrm{SD}\left(\hat{Z}\right)}$$ (10)

where the term $\text{mean}\left(\hat{Z}\right)$ denotes the mean of vector $\hat{Z}$, while $\mathrm{SD}\left(\hat{Z}\right)$ stands for its standard deviation. The significance of the distinction between $\text{mean}\left(\hat{Z}\right)$ and *z* is assessed using the *P* value derived from the *t*-distribution. In this research, the time point *t* is considered as the pre-deterioration state if the SCNE score *H_t_* satisfies two criteria: first, *H_t_* > *H*_*t* − 1_, signifying an upward trend in the score, and second, *H_t_* exhibits statistical significance (*P* < 0.05) in comparison to the prior information. Additional details can be found in section G of the Supplementary Materials.

### Data processing and functional analysis

To illustrate the functionality of the SCNE method, it has been applied to a numerical simulation as well as five real datasets: KIRC, STAD, and LUAD data from the TCGA repository and influenza infection data (accession number: GSE30550) and single-cell data of EPCD in colorectal cancer (accession number: GSE161277) from the Gene Expression Omnibus (GEO) repository. Regarding tumor datasets, both tumor and tumor-adjacent samples were included. Tumor samples were categorized into distinct stages using available stage information, excluding samples with incomplete stage information. The tumor-adjacent samples, which correspond to a comparatively healthy stage, were utilized as the reference group. Additional details on the sampling conditions can be found in section H of the Supplementary Materials. For single-cell colorectal cancer data, Seurat pipelines were employed for the analysis of the single-cell RNA-sequencing data [41]. To address the biological variations among tissues, the R package Harmony was used to implement batch effect correction [42]. In the case of all datasets, we conduct a filtering step that eliminates probes lacking corresponding National Center for Biotechnology Information (NCBI) entrez gene symbols.

Pathway analysis was conducted using the Kyoto Encyclopedia of Genes and Genomes (https://www.kegg.jp). Enrichment analysis was carried out using Metascape [43] and the ClusterProfiler package [44]. Functional outcomes are acquired via web service tools accessible through the Gene Ontology Consortium (http://geneontology.org) and client software provided by Ingenuity Pathway Analysis. The visualization of networks was executed using Cytoscape software (www.cytoscape.org).

## Data Availability

Five real datasets were employed in this study; this included influenza infection (GSE30550) and single-cell data of EPCD in colorectal cancer (GSE161277) sourced from the GEO database (http://www.ncbi.nlm.nih.gov/geo/) and KIRC, STAD, and LUAD data obtained from the TCGA database (http://cancergenome.nih.gov). The source code of the algorithm and related data are available at https://github.com/zhongjiayuan/SCNE_project.

## Supplementary Materials

Supplementary 1 Sections A to H Figs. S1 to S12 Tables S1 and S2 References [1–12]
