# Supplementary material for: Uncovering the Pre-Deterioration State during Disease Progression Based on Sample-Specific Causality Network Entropy (SCNE)
Source: Research (Wash D C). 2024 Apr 29;7:0368. doi: 10.34133/research.0368 (PMC11075703; doi:10.34133/research.0368)
Supplement: Supplementary 1 — Sections A to H Figs. S1 to S12 Tables S1 and S2 References [1–12] [file research.0368.f1.pdf]

Supplementary Information: Uncovering the pre-deterioration state during disease progression based on sample-specific causality network entropy (SCNE)

Contents

A. Description of dynamical system for numerical simulation S2

B. Comparison of the performance among different single-sample detection methods S10

C. Validation of the identified pre-deterioration state S12

D. Intercellular communication probabilities across different cell subset S14

E. Description of constructing the sample-specific causality network S15

F. Description of calculating the expression fluctuation (FT) S16

G. Description of identifying the pre-deterioration state S17

H. Description of the five real-world datasets S18

A. Description of dynamical system for numerical simulation

To further exhibit the effectiveness of our SCNE method, we executed a numerical simulation employing an 18-node regulatory network (illustrated in Figure S1). Such a regulatory network model is commonly used to represent gene regulatory networks in various biological processes [1,2]. This 18-node regulatory network, formulated in Michaelis-Menten configuration, can be described by the ensuing set of 18 differential equations.

$$
\begin{cases}
\frac{dz_1(t)}{dt} = \frac{(8-4|s|)z_2(t)}{15(1+z_2(t))} - \frac{(4+4|s|)}{15} z_1(t) + \zeta_1(t) \\
\frac{dz_2(t)}{dt} = \frac{(4-2|s|)z_1(t)}{15(1+z_1(t))} - \frac{(8+2|s|)z_2(t)}{15(1+z_2(t))} + \zeta_2(t) \\
\frac{dz_3(t)}{dt} = \frac{(4|s|-10)}{15} + \frac{(5-2|s|)}{15(1+z_1(t))} + \frac{(5-2|s|)}{15(1+z_2(t))} - z_3(t) + \zeta_3(t) \\
\frac{dz_4(t)}{dt} = \frac{(12-4|s|)}{15} + \frac{(2|s|-6)}{15(1+z_1(t))} + \frac{(2|s|-6)}{15(1+z_2(t))} - \frac{6}{5} z_4(t) + \zeta_4(t) \\
\frac{dz_5(t)}{dt} = \frac{(4|s|-14)}{15} + \frac{(7-2|s|)}{15(1+z_1(t))} + \frac{(7-2|s|)}{15(1+z_2(t))} - \frac{7}{5} z_5(t) + \zeta_5(t) \\
\frac{dz_6(t)}{dt} = \frac{(4|s|-16)}{15} + \frac{(8-2|s|)}{15(1+z_1(t))} + \frac{(8-2|s|)}{15(1+z_2(t))} - \frac{8}{5} z_6(t) + \zeta_6(t) \\
\frac{dz_7(t)}{dt} = \frac{(18-4|s|)}{15} + \frac{(2|s|-9)}{15(1+z_1(t))} + \frac{(2|s|-9)}{15(1+z_2(t))} - \frac{9}{5} z_7(t) + \zeta_7(t) \\
\frac{dz_8(t)}{dt} = -\frac{2z_1(t)}{15(1+z_1(t))} - \frac{2z_2(t)}{15(1+z_2(t))} - \frac{2z_6(t)}{5(1+z_6(t))} + \frac{2z_{10}(t)}{5(1+z_{10}(t))} + \frac{3z_{12}(t)}{5(1+z_{12}(t))} \\
+ \frac{z_{15}(t)}{5(1+z_{15}(t))} - \frac{z_{16}(t)}{5(1+z_{16}(t))} - 2z_8(t) + \zeta_8(t) \\
\frac{dz_9(t)}{dt} = -\frac{z_1(t)}{5(1+z_1(t))} - \frac{z_2(t)}{5(1+z_2(t))} - \frac{3z_6(t)}{5(1+z_6(t))} - \frac{11}{5} z_9(t) + \zeta_9(t) \\
\frac{dz_{10}(t)}{dt} = \frac{3z_{12}(t)}{5(1+z_{12}(t))} - \frac{12}{5} z_{10}(t) + \zeta_{10}(t) \\
\frac{dz_{11}(t)}{dt} = \frac{z_{12}(t)}{4(1+z_{12}(t))} - \frac{13}{5} z_{11}(t) + \zeta_{11}(t) \\
\frac{dz_{12}(t)}{dt} = \frac{2z_{15}(t)}{5(1+z_{15}(t))} - \frac{2z_{16}(t)}{5(1+z_{16}(t))} - \frac{14}{5} z_{12}(t) + \zeta_{12}(t) \\
\frac{dz_{13}(t)}{dt} = -\frac{z_{15}(t)}{5(1+z_{15}(t))} - \frac{19z_{16}(t)}{5(1+z_{16}(t))} - 5z_{13}(t) + \zeta_{13}(t) \\
\frac{dz_{14}(t)}{dt} = -\frac{4z_{10}(t)}{5(1+z_{10}(t))} - \frac{4z_{12}(t)}{5(1+z_{12}(t))} - \frac{16}{5} z_{14}(t) + \zeta_{14}(t) \\
\frac{dz_{15}(t)}{dt} = \frac{z_{16}(t)}{10(1+z_{16}(t))} - \frac{7}{2} z_{15}(t) + \zeta_{15}(t) \\
\frac{dz_{16}(t)}{dt} = \frac{z_{15}(t)}{10(1+z_{15}(t))} - \frac{7}{2} z_{16}(t) + \zeta_{16}(t) \\
\frac{dz_{17}(t)}{dt} = -\frac{z_{15}(t)}{10(1+z_{15}(t))} + \frac{z_{16}(t)}{10(1+z_{16}(t))} - \frac{19}{5} z_{17}(t) + \zeta_{17}(t) \\
\frac{dz_{18}(t)}{dt} = -\frac{z_{15}(t)}{10(1+z_{15}(t))} + \frac{z_{16}(t)}{10(1+z_{16}(t))} - \frac{z_{17}(t)}{5(1+z_{17}(t))} - 4z_{18}(t) + \zeta_{18}(t)
\end{cases}
\qquad (S1)
$$

where s is a scalar control parameter and $\varsigma_i(t)$ (*i* = 1, 2, ⋯, 18) represents Gaussian noises with zero means and covariances represented by $k_{ij} = Cov(\varsigma_i, \varsigma_j)$. The concentrations of mRNA-i are given by $z_i(t)$ (*i* = 1, 2, ⋯ , 18). In Eq.(S1), the degradation rates of mRNAs are expressed as R=$(\frac{(4+4|s|)}{15}, \frac{(8+2|s|)}{15}, 1, \frac{6}{5}, \frac{7}{5}, \frac{8}{5}, \frac{9}{5}, 2, \frac{11}{5}, \frac{12}{5}, \frac{13}{10}, \frac{14}{10}, 5, \frac{16}{10}, \frac{7}{2}, \frac{7}{2}, \frac{19}{5}, 4)$. $\overline{Z} = (\overline{z_1}, \overline{z_2}, \cdots, \overline{z_{18}}) = (0, 0, \cdots, 0)$ is the stable equilibrium point of the dynamic system described by Eq. (S1). Through application of the Euler scheme, Eq. (S1) can be discretized into the subsequent set of discrete equations, utilizing a small time interval of Δt.

$$
\begin{cases}
z_1(k+1) = z_1(k) + \left[\frac{(8-4|s|)z_2(t)}{15(1+z_2(t))} - \frac{(4+4|s|)}{15} z_1(t) + \zeta_1(t) \right] \Delta t \\
z_2(k+1) = z_2(k) + \left[\frac{(4-2|s|)z_1(t)}{15(1+z_1(t))} - \frac{(8+2|s|)z_2(t)}{15(1+z_2(t))} + \zeta_2(t) \right] \Delta t \\
z_3(k+1) = z_3(k) + \left[\frac{(4|s|-10)}{15} + \frac{(5-2|s|)}{15(1+z_1(t))} + \frac{(5-2|s|)}{15(1+z_2(t))} - z_3(t) + \zeta_3(t) \right] \Delta t \\
z_4(k+1) = z_4(k) + \left[\frac{(12-4|s|)}{15} + \frac{(2|s|-6)}{15(1+z_1(t))} + \frac{(2|s|-6)}{15(1+z_2(t))} - \frac{6}{5} z_4(t) + \zeta_4(t) \right] \Delta t \\
z_5(k+1) = z_5(k) + \left[\frac{(4|s|-14)}{15} + \frac{(7-2|s|)}{15(1+z_1(t))} + \frac{(7-2|s|)}{15(1+z_2(t))} - \frac{7}{5} z_5(t) + \zeta_5(t) \right] \Delta t \\
z_6(k+1) = z_6(k) + \left[\frac{(4|s|-16)}{15} + \frac{(8-2|s|)}{15(1+z_1(t))} + \frac{(8-2|s|)}{15(1+z_2(t))} - \frac{8}{5} z_6(t) + \zeta_6(t) \right] \Delta t \\
z_7(k+1) = z_7(k) + \left[\frac{(18-4|s|)}{15} + \frac{(2|s|-9)}{15(1+z_1(t))} + \frac{(2|s|-9)}{15(1+z_2(t))} - \frac{9}{5} z_7(t) + \zeta_7(t) \right] \Delta t \\
z_8(k+1) = z_8(k) + \left[\frac{2z_1(t)}{15(1+z_1(t))} - \frac{2z_2(t)}{15(1+z_2(t))} - \frac{2z_6(t)}{5(1+z_6(t))} + \frac{2z_{10}(t)}{5(1+z_{10}(t))} + \frac{3z_{12}(t)}{5(1+z_{12}(t))} \right. \\
\left. + \frac{z_{15}(t)}{5(1+z_{15}(t))} - \frac{z_{16}(t)}{5(1+z_{16}(t))} - 2z_8(t) + \zeta_8(t) \right] \Delta t \\
z_9(k+1) = z_9(k) + \left[-\frac{z_1(t)}{5(1+z_1(t))} - \frac{z_2(t)}{5(1+z_2(t))} - \frac{3z_6(t)}{5(1+z_6(t))} - \frac{11}{5} z_9(t) + \zeta_9(t) \right] \Delta t \\
z_{10}(k+1) = z_{10}(k) + \left[\frac{\mathrm{d}z_{10}(t)}{dt} = \frac{3z_{12}(t)}{5(1+z_{12}(t))} - \frac{12}{5} z_{10}(t) + \zeta_{10}(t) \right] \Delta t \\
z_{11}(k+1) = z_{11}(k) + \left[\frac{\mathrm{d}z_{11}(t)}{dt} = \frac{z_{12}(t)}{4(1+z_{12}(t))} - \frac{13}{5} z_{11}(t) + \zeta_{11}(t) \right] \Delta t \\
z_{12}(k+1) = z_{12}(k) + \left[\frac{\mathrm{d}z_{12}(t)}{dt} = \frac{2z_{15}(t)}{5(1+z_{15}(t))} - \frac{2z_{16}(t)}{5(1+z_{16}(t))} - \frac{14}{5} z_{12}(t) + \zeta_{12}(t) \right] \Delta t \\
z_{13}(k+1) = z_{13}(k) + \left[-\frac{z_{15}(t)}{5(1+z_{15}(t))} - \frac{19z_{16}(t)}{5(1+z_{16}(t))} - 5z_{13}(t) + \zeta_{13}(t) \right] \Delta t \\
z_{14}(k+1) = z_{14}(k) + \left[-\frac{4z_{10}(t)}{5(1+z_{10}(t))} - \frac{4z_{12}(t)}{5(1+z_{12}(t))} - \frac{16}{5} z_{14}(t) + \zeta_{14}(t) \right] \Delta t \\
z_{15}(k+1) = z_{15}(k) + \left[\frac{z_{16}(t)}{10(1+z_{16}(t))} - \frac{7}{2} z_{15}(t) + \zeta_{15}(t) \right] \Delta t \\
z_{16}(k+1) = z_{16}(k) + \left[\frac{z_{15}(t)}{10(1+z_{15}(t))} - \frac{7}{2} z_{16}(t) + \zeta_{16}(t) \right] \Delta t \\
z_{17}(k+1) = z_{17}(k) + \left[-\frac{z_{15}(t)}{10(1+z_{15}(t))} + \frac{z_{16}(t)}{10(1+z_{16}(t))} - \frac{19}{5} z_{17}(t) + \zeta_{17}(t) \right] \Delta t \\
z_{18}(k+1) = z_{18}(k) + \left[-\frac{z_{15}(t)}{10(1+z_{15}(t))} + \frac{z_{16}(t)}{10(1+z_{16}(t))} - \frac{z_{17}(t)}{5(1+z_{17}(t))} - 4z_{18}(t) + \zeta_{18}(t) \right] \Delta t
\end{cases}
\qquad (S2)
$$

Where $Z(k)$ corresponds to the vector $Z(t)$ at the time instant $k\Delta t$. The Jacobian matrix of Eq. (S2) is denoted as $J = \left.\frac{\partial f(Z(k);S)}{\partial Z}\right|_{Z=\overline{Z}}$, with

$$J = e^{\Delta t \cdot A} \qquad (S3)$$

By setting $\Delta t = 1$, it becomes possible to deduce eight unique eigenvalues from Eq. (S3). The most prominent eigenvalue meets the condition $0.68^{|s|} \rightarrow 1$ when $S \rightarrow 0$, This behavior signifies that the most substantial eigenvalue of the differential system represented by Eq. (S1) gradually approaches 0 from the negative direction as $S \rightarrow 0$. Consequently, the equilibrium point $\overline{Z}$ is stable if $s \in (0,1]$. This particular parameter $s = 0$ denotes the point of bifurcation, signifying a qualitative shift in the system.

The tipping point in a dynamic system refers to the point of bifurcation where an equilibrium loses its stability. In our model, the parameter denoted as " $s = 0$ " represents this distinct bifurcation point. At this specific point, a bifurcation occurs when $s < 0$, causing all eigenvalues of the Jacobian matrix in Eq. (S1) at the equilibrium to have negative real parts. This signifies the initial stability of the equilibrium point . As $S \rightarrow 0$, the largest real part (λ) among the eigenvalues gradually approaches 0 and eventually becomes positive after surpassing the bifurcation point. Consequently, the originally stable equilibrium point $\overline{Z} = (\overline{z_1}, \overline{z_2}, \overline{z_3}, ..., \overline{z_{18}})$ becomes unstable, leading to a qualitatively different periodic solution. This bifurcation phenomenon reflects a phase transition in the system. Using the theoretical model in Eq. (S2), we generated numerical simulation datasets for the 18-node network by varying the parameter from -0.5 to 0.15.

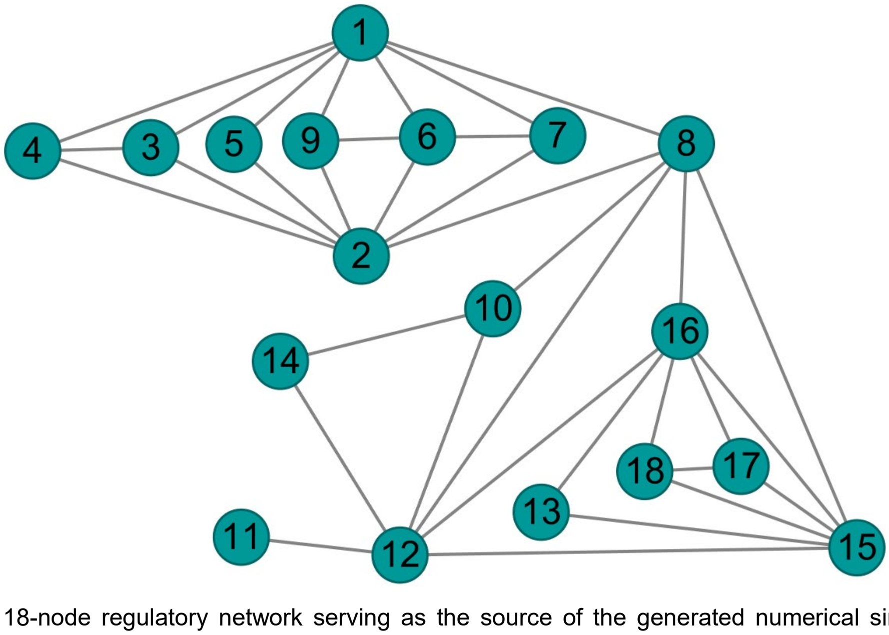

Figure S1. an 18-node regulatory network serving as the source of the generated numerical simulations. This regulatory network is represented using Michaelis-Menten equations and can be described by stochastic differential equations as depicted in Eq. (S1).

Besides, a 6-node regulatory network controlled by a system of stochastic differential equations, as shown in Eq. (S4), was applied to generate simulated data for demonstrating the dynamic changes of DNB (nodes 1, 2, and 3) and non-DNB (nodes 4, 5, and 6) in terms of the expression fluctuation (FT) and causal strength (*w* value).

$$\begin{cases} \frac{\mathrm{d}z_1(t)}{dt} = \frac{(5-2|s|)z_2(t)}{5(1+z_2(t))} + \frac{(5-2|s|)z_3(t)}{5(1+z_3(t))} - \frac{2|s|}{5}z_1(t) + \zeta_1(t) \\ \frac{\mathrm{d}z_2(t)}{dt} = \frac{(2-|s|)z_1(t)}{5(1+z_1(t))} + \frac{(2-|s|)z_3(t)}{5(1+z_3(t))} - \frac{2+|s|}{5}z_2(t) + \zeta_2(t) \\ \frac{\mathrm{d}z_3(t)}{dt} = \frac{(2|s|-5)}{5} + \frac{(2-|s|)}{5(1+z_1(t))} + \frac{(3-|s|)}{5(1+z_2(t))} - \frac{7-|s|}{5}z_3(t) + \zeta_3(t) \\ \frac{\mathrm{d}z_4(t)}{dt} = -\frac{1}{2} + \frac{1}{10(1+z_2(t))} + \frac{1}{10(1+z_3(t))} + \frac{3z_5(t)}{10(1+z_5(t))} + \frac{3}{10(1+z_6(t))} - \frac{8}{5}z_4(t) + \zeta_4(t) \\ \frac{\mathrm{d}z_5(t)}{dt} = \frac{z_6(t)}{10(1+z_6(t))} - \frac{21}{10}z_5(t) + \zeta_5(t) \\ \frac{\mathrm{d}z_6(t)}{dt} = \frac{z_5(t)}{10(1+z_5(t))} - \frac{21}{10}z_6(t) + \zeta_6(t) \end{cases} \quad (S4)$$

where s is a scalar control parameter, and $\zeta_i(t)$ (i = 1, 2,⋯,6) denotes Gaussian noises with zero means and covariances denoted by $k_{ij} = Cov(\varsigma_i, \varsigma_j)$. The concentrations of mRNA-i are indicated as $z_i(t)$. In Eq. (S4), the degradation rates of mRNAs are represented as $\mathrm{R}=(\frac{2|s|}{5}, \frac{2+|s|}{5}, \frac{7-|s|}{5}, \frac{8}{5}, \frac{21}{10}, \frac{21}{10})$. $\overline{Z} = (\overline{z_1}, \overline{z_2}, \overline{z_3}, \cdots, \overline{z_6}) = (0,0,0,\cdots,0)$ is the stable equilibrium point of the dynamic system described by Eq. (S4). By employing the Euler scheme, Eq. (S4) can be transformed into a subsequent set of discrete equations using a small-time interval Δt.

$$\begin{cases} z_1(k+1) = z_1(k) + \left[\frac{(5-2|s|)z_2(t)}{5(1+z_2(t))} + \frac{(5-2|s|)z_3(t)}{5(1+z_3(t))} - \frac{2|s|}{5}z_1(t) + \zeta_1(t) \right]\Delta t \\ z_2(k+1) = z_2(k) + \left[\frac{(2-|s|)z_1(t)}{5(1+z_1(t))} + \frac{(2-|s|)z_3(t)}{5(1+z_3(t))} - \frac{2+|s|}{5}z_2(t) + \zeta_2(t) \right]\Delta t \\ z_3(k+1) = z_3(k) + \left[\frac{(2|s|-5)}{5} + \frac{(2-|s|)}{5(1+z_1(t))} + \frac{(3-|s|)}{5(1+z_2(t))} - \frac{7-|s|}{5}z_3(t) + \zeta_3(t) \right]\Delta t \\ z_4(k+1) = z_4(k) + \left[-\frac{1}{2} + \frac{1}{10(1+z_2(t))} + \frac{1}{10(1+z_3(t))} + \frac{3z_5(t)}{10(1+z_5(t))} + \frac{3}{10(1+z_6(t))} - \frac{8}{5}z_4(t) + \zeta_4(t) \right]\Delta t \\ z_5(k+1) = z_5(k) + \left[\frac{z_6(t)}{10(1+z_6(t))} - \frac{21}{10}z_5(t) + \zeta_5(t) \right]\Delta t \\ z_6(k+1) = z_6(k) + \left[\frac{z_5(t)}{10(1+z_5(t))} - \frac{21}{10}z_6(t) + \zeta_6(t) \right]\Delta t \end{cases} \quad (S5)$$

with a small time Δt, $Z(k)$ corresponds to the vector $Z(t)$ interval at the time instant $k\Delta t$. The Jacobian

matrix of Eq.(S5) is represented as $J = \left.\frac{\partial f(Z(k);S)}{\partial Z}\right|_{Z=\bar{Z}}$, with

$$J = e^{\Delta t \cdot A} \qquad (S6)$$

By setting $\Delta t = 1$, it becomes feasible to derive eight distinct eigenvalues from Eq. (S6). The predominant eigenvalue adheres to the condition $0.67^{|s|} \rightarrow 1$ as $s \rightarrow 0$, This pattern suggests that the most significant eigenvalue of the differential system outlined in Eq. (S4) gradually converges to 0 from the negative direction as $s \rightarrow 0$. Thus, the stability of the equilibrium point $\bar{Z}$ is ensured if $s \in (0,1]$. This specific parameter $s = 0$ signifies the bifurcation threshold, indicating a qualitative transformation in the system.

The disease progression can be divided into three distinct states: a stable relatively normal state, a pre-deterioration state (critical point), and another stable deteriorated state (Figure S2). To clarify the relationship between SCNE and traditional DNB method, a more detailed explanation and corresponding analytical demonstration are described as follows. When a complex system is near the critical point, the traditional DNB method was utilized to quantitatively identify the pre-deterioration state by applying the following two statistical conditions of DNB molecules [3]: (i) The standard deviation (SD) for DNB molecules drastically increases (Figure S3A); and (ii) The Pearson correlation coefficient (PCC) between DNB molecules rapidly increases (Figure S3B). These two properties can also be approximately stated as: the presence of a group of molecules whose expressions are strongly fluctuating and highly correlated at a network level implies an imminent critical transition [4]. Clearly, the traditional DNB method is limited in its focus on undirected networks and only captures dynamic changes in correlations, thus lacking the ability to discern alterations in regulatory relationships. Additionally, it fails to identify DNB genes located at the "central position" of the network. In contrast, our proposed SCNE addresses these limitations of the traditional DNB method by leveraging the expression fluctuation of DNB molecules and their causal regulatory relationships. Specifically, as the system approaches critical state, our SCNE method was developed to quantitatively detect the pre-deterioration state based on the following two criteria: (1) The expression fluctuation (FT) of DNB molecules drastically increases (Figure S3C); and (2) The causal strength (w value) between DNB molecules rapidly increases (Figure S3D). Therefore, our proposed SCNE approach can detect early warning signals for critical transitions or tipping points by quantifying the FT and w value (Figure S3E). Additionally, it exhibits a positive correlation with the composite index (SD*PCC) of the traditional DNB method (Figure S3F).

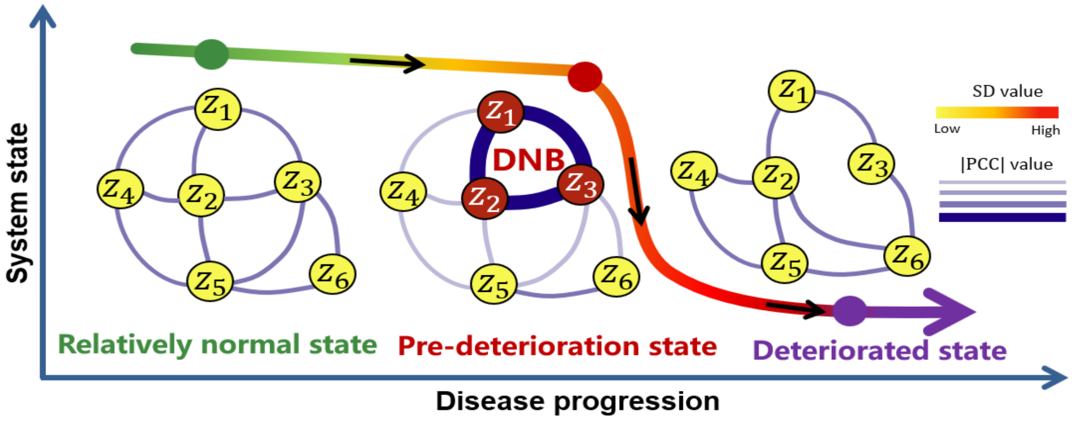

Figure S2. The progression of complex diseases is modeled as three states: a stable relatively normal state, a pre-deterioration state (critical point), and another stable deteriorated state. As the system approaches the critical point, the SD of DNB molecules undergoes a significant increase; and while the PCC among DNB molecules demonstrates a rapid increase.

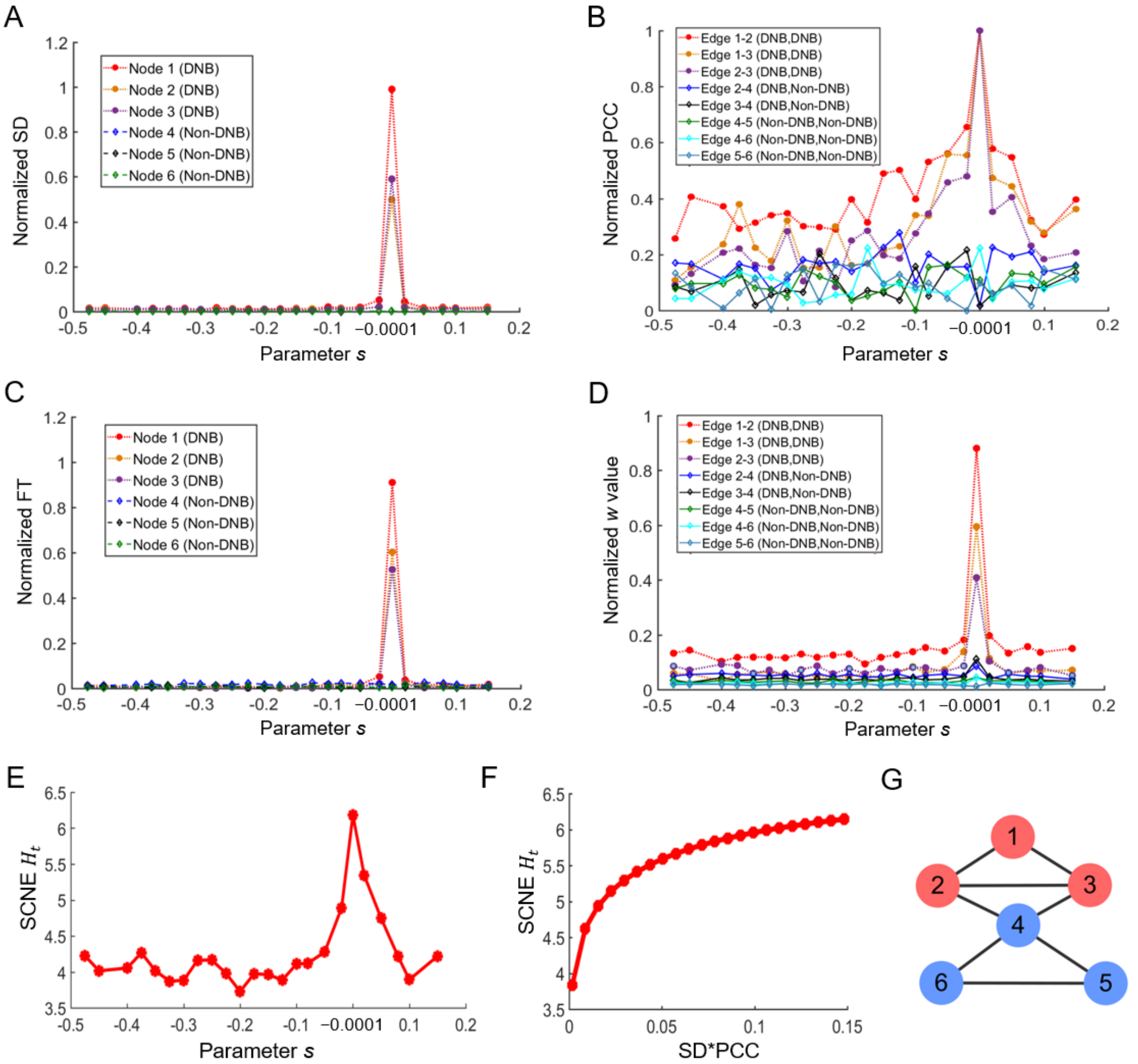

Figure S3. (A)-(B) The curves representing the standard deviation (SD) and Pearson correlation coefficient (PCC) against the parameter s clearly indicate the patterns observed across different nodes. Particularly noteworthy is the sharp increase in deviations (SD(Node 1), SD(Node 2), and SD(Node 3)) and correlations (|PCC(Node 1, Node 2)|, |PCC(Node 1, Node 3)|, and |PCC(Node 2, Node 3)|) as the system approaches the tipping point. (C)-(D) The curves of expression fluctuation (FT) and causal strength (w value) against the parameter s clearly reveal the patterns observed in different nodes. Notably, the fluctuations (FT(Node 1), FT(Node 2), and FT(Node 3)) and correlations (w(Node 1, Node 2), w(Node 1, Node 3), and w(Node 2, Node 3)) experience a sharp increase as the system approaches the tipping point. (E) The curve depicting the SCNE H_t exhibits a sudden increase in the vicinity of the critical point. (F) The SCNE H_t exhibits a positive correlation with the composite index (SD*PCC) of the traditional DNB method. (G) A network comprising six nodes, governed by a model represented in Michaelis-Menten form, serves as the foundation for conducting numerical simulations.

Moreover, we analyzed the relationship between the pivotal indices of the traditional DNB method

and those of our SCNE index. As shown in (Figure S4A), a positive relationship is observed between SD and FT, suggesting that as the SD of DNB increases, its FT also increases. Similarly, there is evidence of a positive correlation between PCC and w value (Figure S4B), indicating that an increase in the w value coincides with a rise in PCC among DNB members.

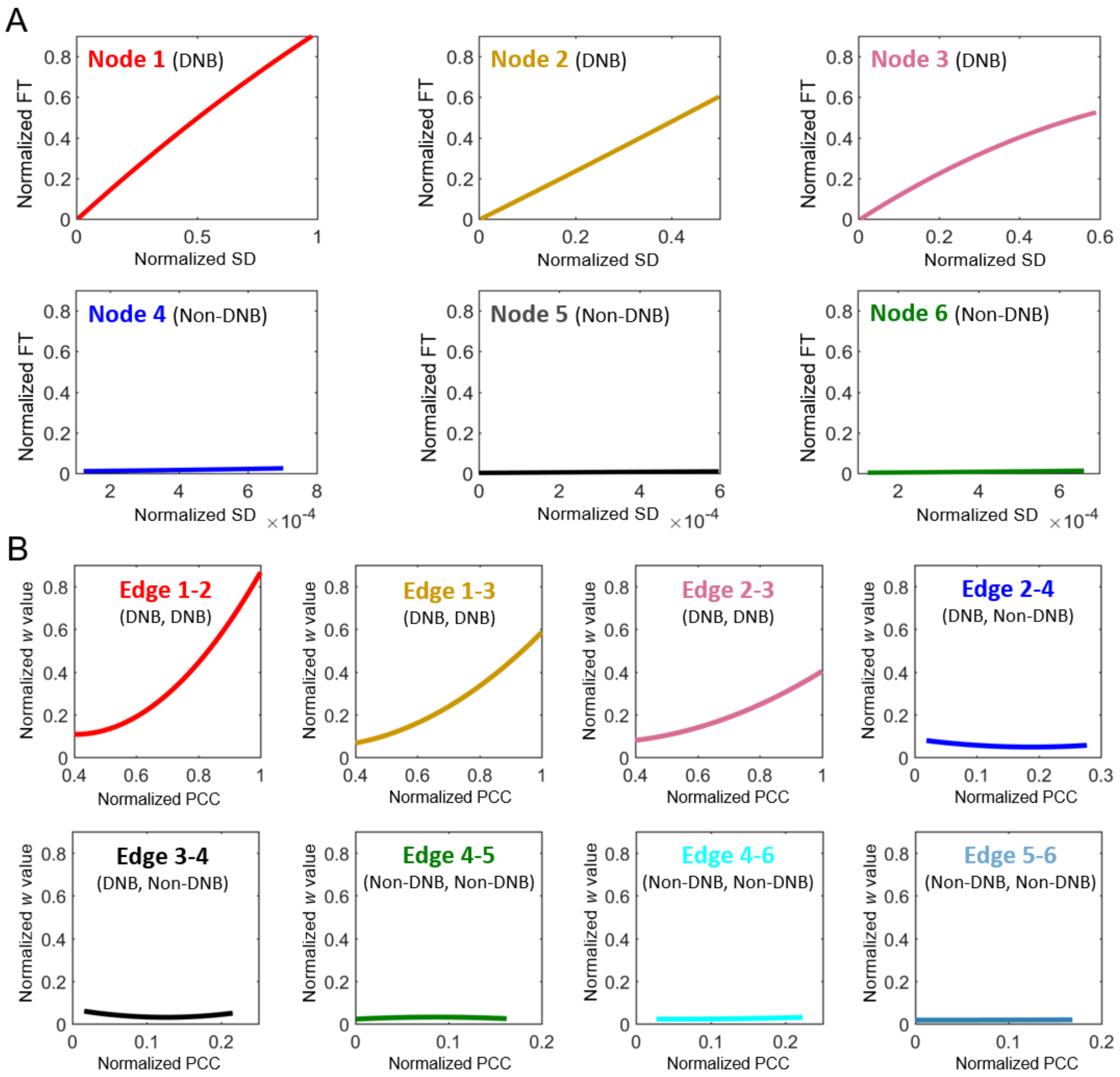

Figure S4. (A) The expression fluctuation (FT) of DNB shows a positive correlation with its standard deviation (SD). (B) Similarly, the causal strength (w value) demonstrates a positive correlation with Pearson correlation coefficient (PCC) among DNB members.

Besides, as depicted in Figure S5, it is evident that as the system approaches the pre-deterioration state, a distinct alteration in the structure of the subnetwork consisting of DNB members (i.e., nodes 1, 2, and 3) signifies an imminent shift in the state of the network. Furthermore, it can be seen from Tables S1 and S2, the expression deviation or fluctuation (FT) of DNB members significantly increases, accompanied by a rapid rise in the causal strength (w value) between them.

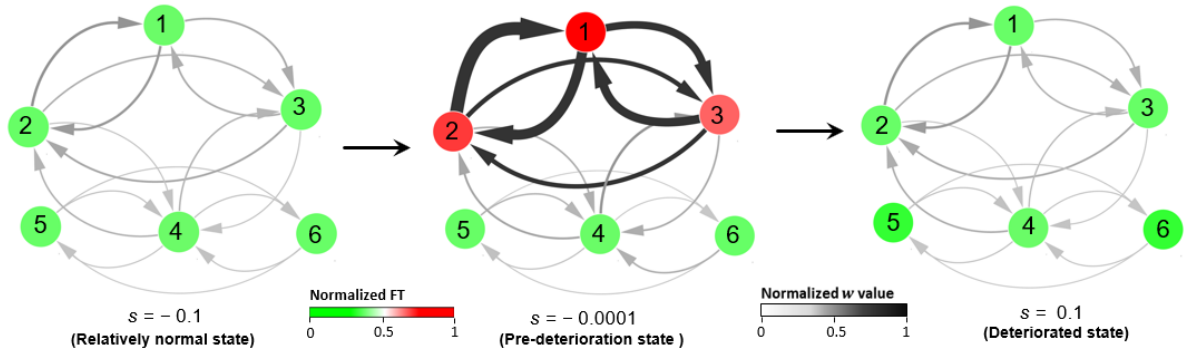

Figure S5. The dynamic evolution of the regulatory network uncovers a notable alteration in the structure of the subnetwork comprised of DNB members near the pre-deterioration state.

Table S1. DNB and non-DNB of dynamic changes in the expression fluctuation (FT) at different states during the progression of the disease

Nodes / Parameters	Node 1 DNB	Node 2 DNB	Node 3 DNB	Node 4 Non-DNB	Node 5 Non-DNB	Node 6 Non-DNB
$s = -0.1$ Relatively normal state	0.018	0.008	0.0069	0.02	0.0087	0.01
$s = -0.0001$ Pre-deterioration state	0.89	0.61	0.58	0.015	0.0072	0.0049
$s = 0.1$ Deteriorated state	0.0097	0.0099	0.0068	0.016	0.0025	0.0045

Table S2. DNB and non-DNB of dynamic changes in the causal strength (w value) at different states during the progression of the disease

Parameters / Directed edge	$s = -0.1$ Relatively normal state	$s = -0.0001$ Pre-deterioration state	$s = 0.1$ Deteriorated state
Node 2→1 (DNB, DNB)	0.15	0.91	0.14
Node 3→1 (DNB, DNB)	0.078	0.64	0.063
Node 1→2 (DNB, DNB)	0.129	0.76	0.127
Node 3→2 (DNB, DNB)	0.091	0.43	0.087
Node 4→2 (non-DNB, DNB)	0.063	0.099	0.076
Node 1→3 (DNB, DNB)	0.087	0.55	0.07
Node 2→3 (DNB, DNB)	0.069	0.41	0.073
Node 4→3 (non-DNB, DNB)	0.06	0.14	0.045
Node 2→4 (DNB, non-DNB)	0.023	0.074	0.021
Node 3→4 (DNB, non-DNB)	0.022	0.08	0.023
Node 5→4 (non-DNB, non-DNB)	0.028	0.058	0.03
Node 6→4 (non-DNB, non-DNB)	0.026	0.072	0.024
Node 4→5 (non-DNB, non-DNB)	0.023	0.029	0.029
Node 6→5 (non-DNB, non-DNB)	0.014	0.016	0.017
Node 4→6 (non-DNB, non-DNB)	0.027	0.013	0.023
Node 5→6 (non-DNB, non-DNB)	0.021	0.0078	0.017

B. Comparison of the performance among different single-sample detection methods

Many single-sample methods such as single-sample landscape entropy (SLE) [5], and single-sample network module biomarkers (sNMB) [6], and single-sample-based hidden Markov model (sHMM) [7], temporal network flow entropy (TNFE) [8], personalized dynamic network biomarker (PDNB) [9] and landscape dynamic network biomarker (LDNB) [10], have been employed to quantify the criticality of complex diseases on the basis of individual sample/cell. To demonstrate the superiority of our proposed SCNE method in three TCGA dataset, we compared it with these approaches. As shown in Figure S6 and Table 1, our proposed approach exhibits more significant p-values at critical points or identify pre-deterioration states that align better with biological significance, which demonstrates it has better performance in identifying critical signals or pre-deterioration states during disease progression.

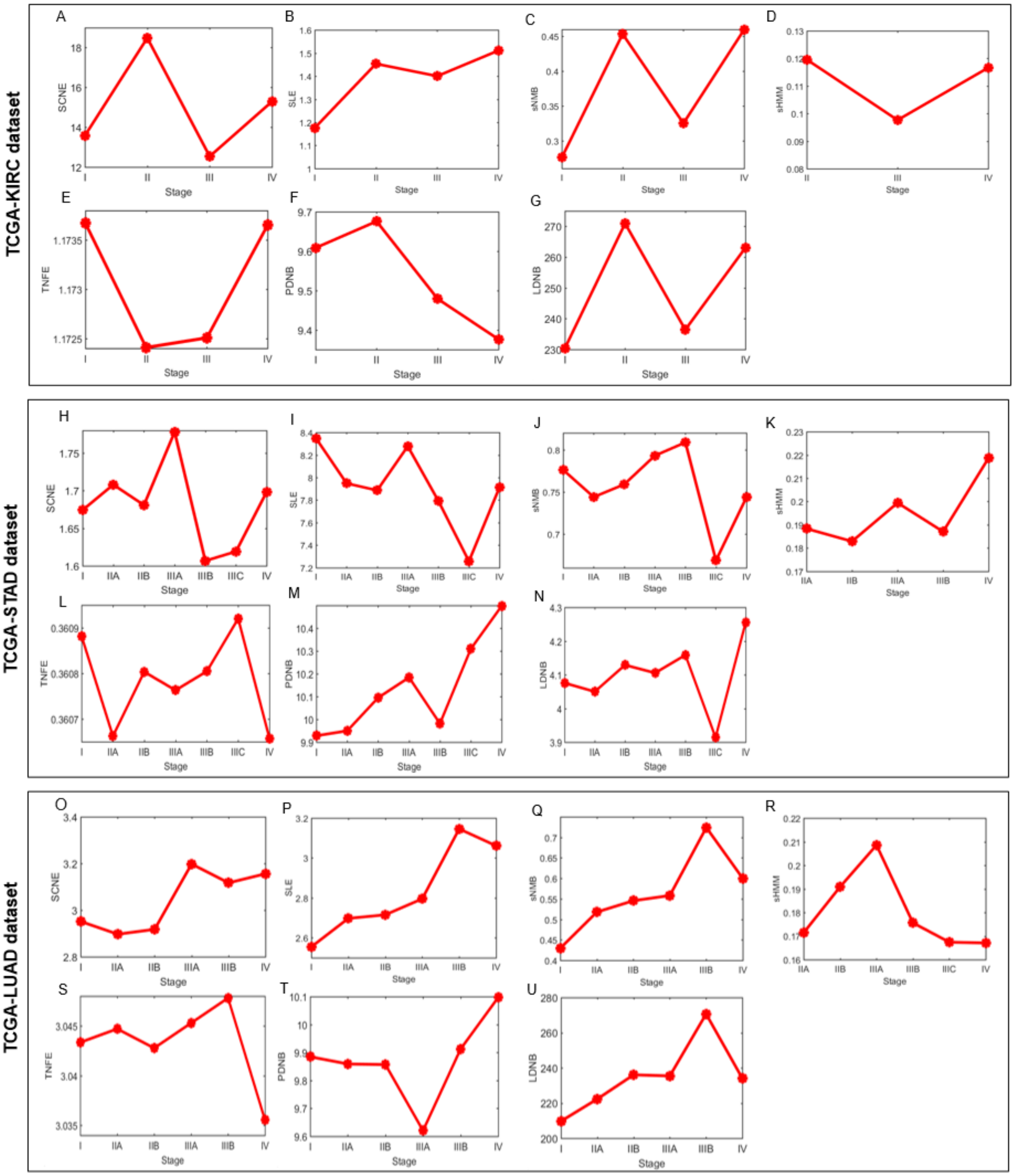

Figure S6. Comparison of dynamic changes performance among different single-sample detection methods. Different single-sample detection methods, including SCNE, SLE, sNMB, sHMM, TNFE, PDNB, and LDNB, are applied to compare the effects across three tumor datasets: (A)-(G) KIRC, (H)-(N) STAD, and (O)-(U) LUAD.

A comparative analysis was carried out between our proposed method (SCNE) and the SNE method utilizing the background protein-protein interaction (PPI) network without the introduced distinctions. For KIRC and STAD data, we conducted a comparative analysis between SCNE and an alternative method that does not incorporate the in-degree and out-degree distinctions. As illustrated in Figure S7, the signal provided by our proposed SCNE method is significantly stronger than that from the alternative method. Therefore, in this study, we leveraged a network that distinguishes between in-degree and out-degree to generate the SCNE score, showcasing improved performance in analyzing the dynamic changes of the network.

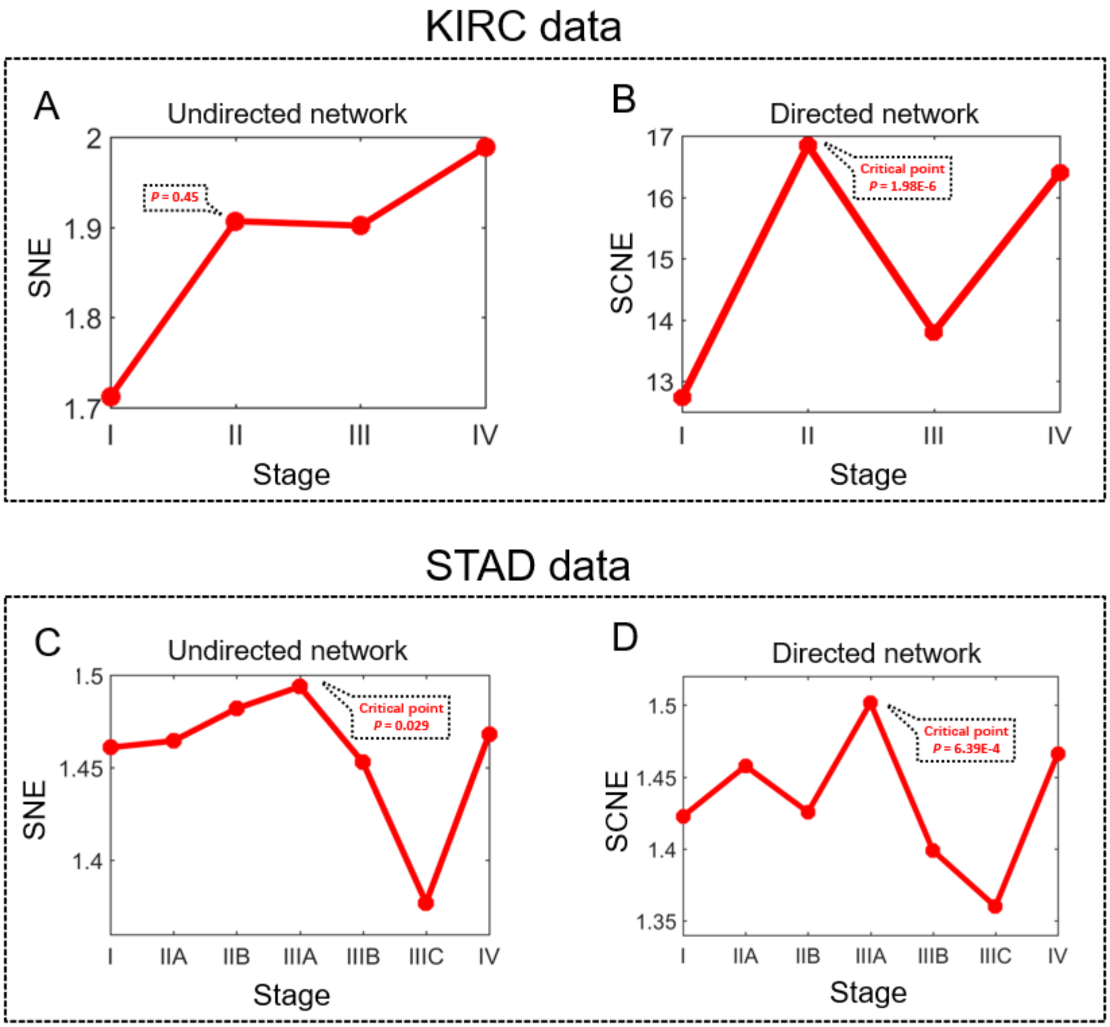

Figure S7. Comparison of dynamic changes performance between the proposed SCNE approach and SNE method which does not incorporate the in-degree and out-degree distinctions. The performance of the two methods is evaluated for the (A)-(B) KIRC dataset. It is evident that no signal is detected using the SNE method, while a significant increase (with a P-value of 1.98E-6) is observed with the SCNE method. Similarly, a comparison of dynamic change performance is conducted for the (C)-(D) STAD dataset. The signal obtained from the SCNE method (with a P-value of 6.39E-4) is notably more significant compared to that from the SNE method (with a P-value of 0.029).

Our proposed SCNE method is data-driven, and its applicability is delineated by three aspects: (1) System characteristics: SCNE can be applied to the analysis of catastrophic systems where abrupt transitions occur, such as ecological systems undergoing regime shifts. Its capacity to capture nuanced temporal patterns leading up to such sudden, catastrophic shifts or events makes it an effective tool for understanding and predicting system dynamics during critical phases. (2) Network architecture considerations: The availability of a species-specific protein-protein interaction (PPI) network or a predefined network structure is necessary for SCNE. This network serves as the foundation for the approach, enabling the incorporation of individualized gene expression data to infer sample-specific causal networks. Therefore, SCNE is applicable to systems where PPI networks or predefined network structures are accessible, as these facilitate the integration of network information into the analysis. (3) Data prerequisites: SCNE necessitates samples obtained from critical states to effectively predict pre-deterioration states. This implies that SCNE's applicability relies on the availability of relevant data collected during critical periods. Without such data, SCNE may not be able to accurately capture the temporal patterns leading up to catastrophic events. In summary, the proposed SCNE is suitable for analyzing catastrophic systems equipped with available PPI networks or predefined network structures, and critical state samples for predictions of pre-deterioration states. We appreciate your insightful comments, and these revisions aim to provide a more comprehensive understanding of the SCNE method.

However, limitations of our SCNE method include its dependency on a PPI network as the background network and the necessity for a reference group composed of relatively healthy samples. Specifically, the construction of the sample-specific causality network is based on a priori knowledge-based PPI network. It is emphasized that reference samples should be chosen from normal tissues or (relatively) healthy individuals within the same tissue/organ. Additionally, it may face challenges in identifying multi-stage deteriorations during a cascade deterioration process of a complex disease for an individual. Therefore, its applicability is delineated by three specific requirements. Firstly, the system should have access to stable samples representing a relatively normal or stable state, which are essential for training the regression equation. Secondly, the availability of a species protein-protein interaction (PPI) network or a predefined network structure is necessary to serve as the background for the approach. Lastly, the system should possess samples obtained from critical states to facilitate effective predictions of the pre-deterioration state. In summary, the proposed SCNE is suitable for systems equipped with stable training samples, a known network structure, and critical state samples for predictions of pre-deterioration states.

C. Validation of the identified pre-deterioration state

The proposed method has been employed to three distinct TCGA datasets: KIRC, STAD, and LUAD. The results presented in Figs 3A-C of the main text demonstrate that the SCNE method successfully identifies the pre-deterioration state prior to lymph node metastasis (stage II) for KIRC, and the pre-deterioration state prior to distant metastasis (stage IIIA) for STAD and LUAD. To validate the identified

pre-deterioration state, survival times are compared between samples before and after the pre-deterioration stage using Kaplan-Meier log-rank analysis. Two criteria are used for validation: (1) samples derived before the identified pre-deterioration stage exhibit higher survival rates compared to those after the stage, and (2) the survival time of samples from the identified pre-deterioration stage is significantly longer ($p < 0.05$) than that of samples from the subsequent stage (Figure S8).The analysis reveals that the survival time of samples after the pre-deterioration stage is significantly longer than that of samples before the pre-deterioration stage (Figure S9). For KIRC, a statistical difference ($p < 0.0001$) is observed between the survival curves of samples before and after stage II (Figure S9A). Moreover, as depicted in Figure S9B, the survival time of patients from stage II (the pre-deterioration state) is considerably longer compared to the subsequent stage III. In the case of STAD, Figure S9C indicates that the survival time of samples before stage IIIA is significantly ($p = 0.001$) longer than that after stage IIIA. Additionally, the survival time of samples from stage IIIA (the critical point) is significantly different ($p = 0.041$) from that of stage IIIB samples (Figure S9D). For LUAD, the survival curve before stage IIIA (the pre-deterioration state) is significantly different ($p < 0.0001$) from the curve after stage IIIA (Figure S9E). The survival time of patients from stage IIIA is significantly ($p = 0.032$) longer than that of stage IIIB samples (Figure S9F). These results demonstrate the ability of the SCNE method to detect early-warning signals indicating a critical transition in patient survival.

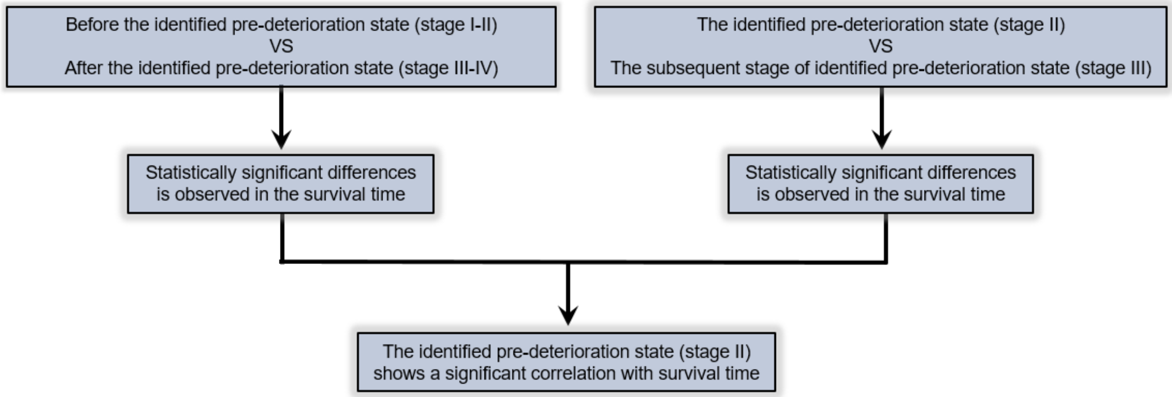

Figure S8. an illustrative process for validation of the identified pre-deterioration state.

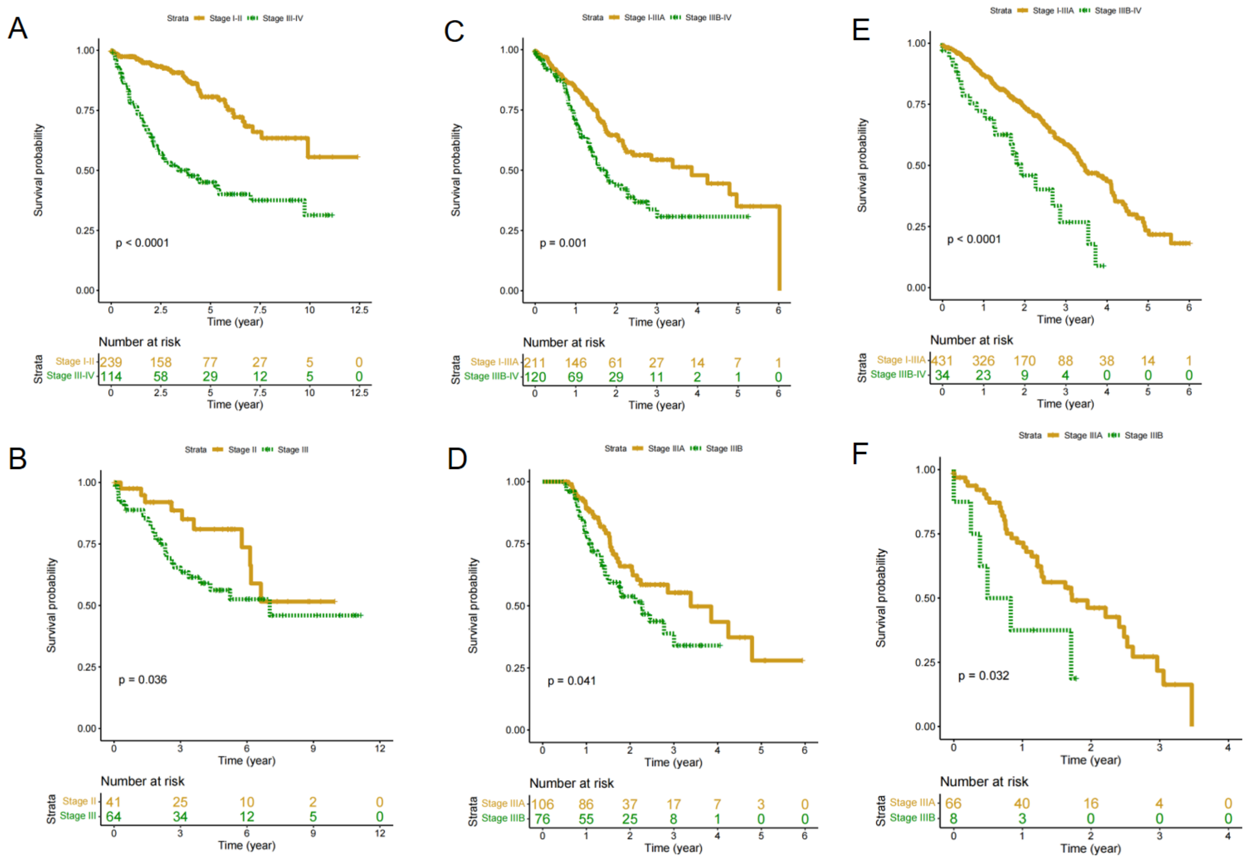

Figure S9. Survival analysis before and after the identified pre-deterioration state in three distinct TCGA datasets: (A)&(B) KIRC, (C)&(D) STAD, and (E)&(F) LUAD.

D. Intercellular communication probabilities across different cell subset

In the context of the TGF-β signaling pathway, the MMP7+ FABP1+ TFF1+ CKB+ epithelial cell subset (Cluster 6) and the macrophage subset communicate a potent proliferation signal to the FABP5+ S100P+ PLA2G2A+ TUBA1B+ epithelial cell subset (Cluster 4). This communication pattern potentially suggests an imminent exacerbation in the decline of epithelial cells. Moreover, within the tumor microenvironment (TME), macrophages exhibit the highest receptor-ligand communication probability in the TGF-β signaling pathway (Figure S10), indicating their potential contribution to the propagation of epithelial cell deterioration.

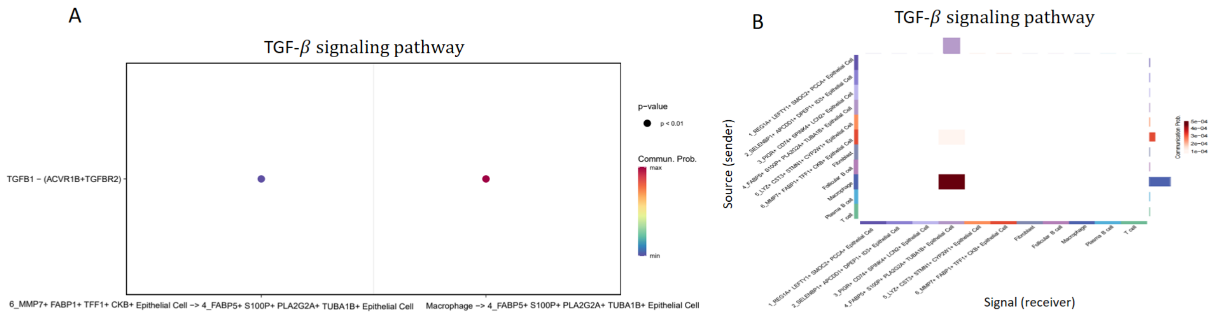

Figure S10. (A)-(B) Intercellular communication probabilities across different cell subpopulations based on the TGF-β signaling pathway.

E. Description of constructing the sample-specific causality network

The sample-specific causality network can be constructed by the case sample at time point t, as depicted in Figure S11.

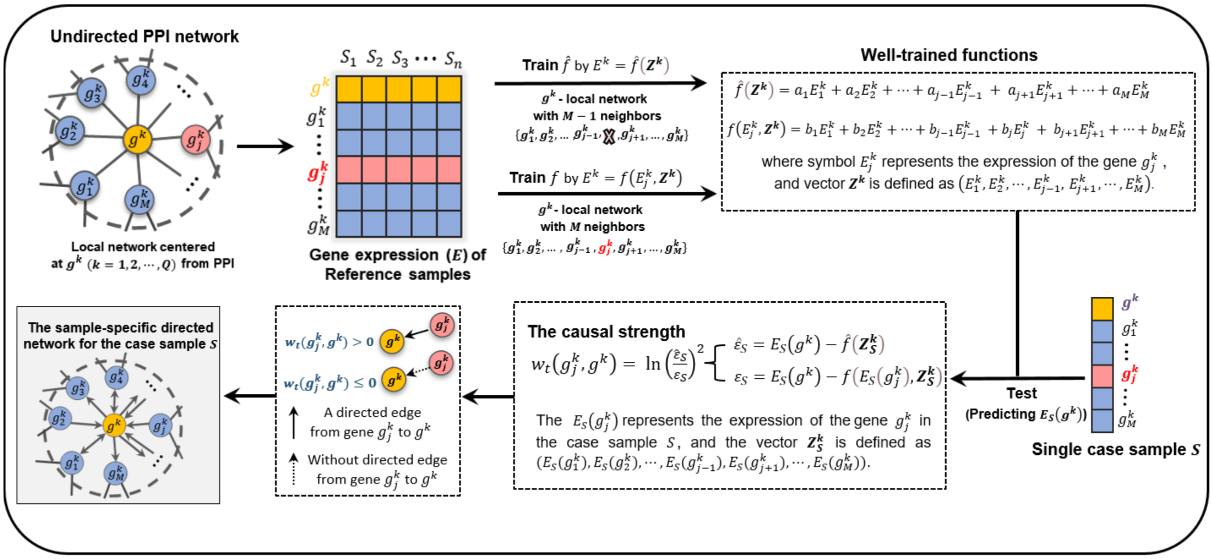

Figure S11: An illustrative process for constructing the sample-specific causality network.

Here's a step-by-step explanation:

1. Initiation: For a local network N^k centred with a gene g^k in the protein-protein interaction (PPI) network, whose 1st-order neighbours are genes $\{g_1^k, g_2^k, \cdots, g_{j-1}^k, g_j^k\ \ g_{j+1}^k, \cdots, g_M^k\}$, we assume that all the 1st-order neighbors g_i^k $(i = 1,2,\cdots,M)$ are the cause of the center node g^k, i.e., the change of expressions of any neighbor g_j^k may affect that of g^k. A group of relative healthy reference samples serves as the training samples to determine regression models $\hat{f}$ and f, while a single case sample at each time point t is designated as the test sample. By inputting the test sample into $\hat{f}$ and f, respectively, the output $\hat{\varepsilon} = E^k - \hat{f}(\mathbf{Z}^k)$ and $\varepsilon = E^k - f(E_j^k, \mathbf{Z}^k)$ are obtained to infer the sample-specific causality network.

2. Detection Framework: Two contrasting models, H0 (null hypothesis without causality) and H1 (alternative hypothesis with causality), are established:

- H0 Model: To assess the causal influence of a neighbor node g_j^k on the central node g^k, we remove g_j^k from the g^k-local network. Subsequently, we perform regression analysis on the expression E^k of g^k using the expressions from all remaining neighbors in the training samples to derive a regression equation $\hat{f}$. Then, the output error $\hat{\varepsilon}$ can be obtained by inputting the test case sample into $\hat{f}$.
- H1 Model: In contrast, this model includes g_j^k in the local network, and regression analysis is

performed on the expression E^k of g^k using the expressions from all its neighbors to obtain a regression equation f. Moreover, the new output error ε can be calculated by implementing the test case sample into f.

3. Causal Strength Evaluation: The squared errors ε^2 and $\hat{\varepsilon}^2$ are employed to infer potential causal regulation from g_j^k to g^k. If ε^2 is less than $\hat{\varepsilon}^2$, it suggests that g_j^k contributes to accurately predicting g^k in the test sample, indicating a causal link between g_j^k and g^k. Conversely, if ε^2 is greater than $\hat{\varepsilon}^2$, g_j^k is considered unsuitable for predicting g^k, implying that g_j^k is not the cause of g^k in this case. Thus, the causal strength $w_t(g_j^k, g^k) = \ln\left(\frac{\hat{\varepsilon}}{\varepsilon}\right)^2$ is utilized to quantify the causality from g_j^k to g^k, where a positive value indicates causality and a non-positive value indicates no causality.

4. Inference of Sample-Specific Causality Network: For the single case sample S at each time point t, by inputting it into the trained $\hat{f}$ and f, respectively, we can obtain output errors $\hat{\varepsilon}_S = E_S(g^k) - \hat{f}(\boldsymbol{Z_S^k})$ and $\varepsilon_S = E_S(g^k) - f(E_S(g_j^k), \boldsymbol{Z_S^k})$. Here, the symbol $E_S(g_j^k)$ represents the expression of the j-th gene of g^k -local network in the single case sample S, and the vector $\boldsymbol{Z_S^k}$ is represented as $(E_S(g_1^k), E_S(g_2^k), \cdots, E_S(g_{j-1}^k), E_S(g_{j+1}^k), \cdots, E_S(g_M^k))$. Moreover, the causal strength $w_t(g_j^k, g^k) = \ln\left(\frac{\hat{\varepsilon}_S}{\varepsilon_S}\right)^2$ is employed to infer potential causality from g_j^k to g^k in the case sample S. After evaluating each neighbor node g_j^k in the local network of g^k using the $w_t(g_j^k, g^k)$, we can derive the causal subnetwork centered at node g^k. Therefore, this process allows us to infer the sample-specific causality network for the case sample at each time point.

F. Description of calculating the expression fluctuation (FT)

The calculation for expression fluctuation (FT) is outlined as follows. Specifically, given n reference samples, the distribution of each gene's expression can be obtained as its reference distribution [11,12]. The expression of the gene in a new sample (e.g., a case sample for statistical testing) can be compared with its reference distribution to estimate the deviation of its expression from the reference samples (n samples). Therefore, the expression deviation of a gene in the case sample can be expressed as the deviation from expectation based on its reference distribution (Figure S12). Specifically, the expression deviation of a gene in a case sample against n reference samples, i.e., the expression fluctuation (FT) for gene g^k, can be defined as:

$$\mathrm{FT}(g^k) = \left|\frac{E_S(g^k) - \mu(E_{\mathrm{re}}(g^k))}{\sigma(E_{\mathrm{re}}(g^k))}\right| \tag{S7}$$

where $E_s(g^k)$ is the expression of gene g^k in the new sample S, $\mu(E_{\mathrm{re}}(g^k))$ is the average expression of gene g^k in reference samples, and $\sigma(E_{\mathrm{re}}(g^k))$ is the expression deviation of gene g^k in reference samples.

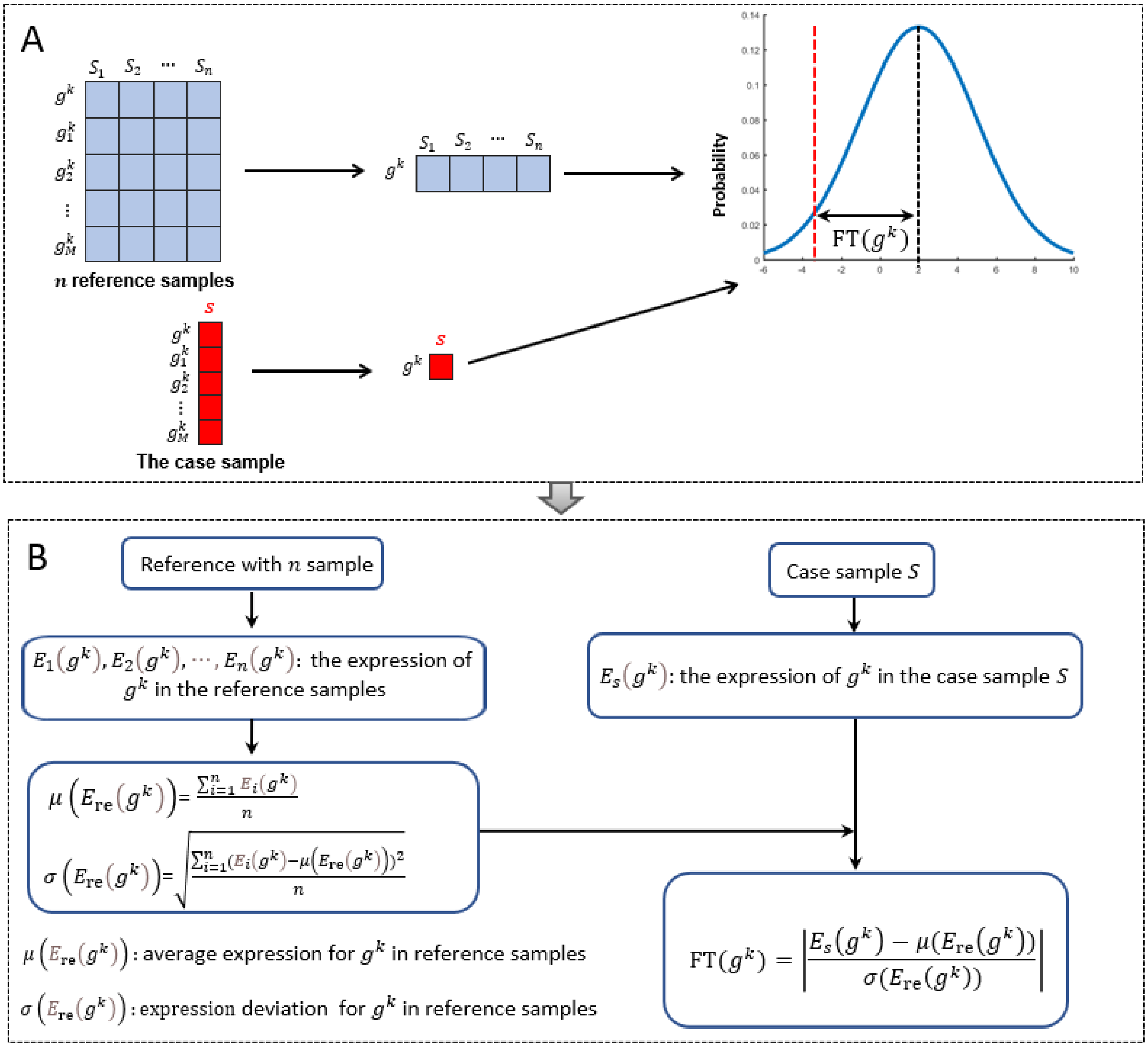

Figure S12. The expression distribution of each gene can be obtained from the reference samples, while the expression fluctuation (FT) of a gene in the case sample can be expressed as the deviation from expectation based on its reference distribution.

G. Description of identifying the pre-deterioration state

The H_t index integrates critical properties of the DNB biomolecules, including the expression fluctuation of nodes and the causal strength variation among them. Specifically，as shown in Figure S4, when the system approaches pre-deterioration state, the DNB members satisfy the following two criteria based on the observed data: (1) The causal strength (w value) between DNB members rapidly increases; and (2) The expression deviation or fluctuation (FT) of DNB members drastically increases. Thus, for the sub-network composed of some variables (DNB members), a noticeable change in its network structure occurs when the system is near a critical state, indicating an imminent critical transition. By exploring the dynamic information of such a group of dominant variables at a network level, it becomes possible to predict the qualitative state transition. Consequently, the H_t index is designed to quantify the

expression fluctuation and causal strength variation triggered by each single sample against a group of given reference samples, providing the warning signals of the pre-deterioration state.

In the context of biological systems, H_t encompasses two key aspects of information. Firstly, it involves the dynamic fluctuations of molecules, and secondly, it explores the regulatory relationships among these molecules. As depicted in Figure 5F in the main text, before and after the pre-deterioration state, the identified signaling molecules exhibit dynamic changes in their expression and consequently exerts effects on the expression variations of their neighboring genes, revealing the underlying regulatory mechanism associated with crucial biological processes.

In order to evaluate the capability of the SCNE score in measuring critical behavior, we employ a one-sample t-test to determine if there is a statistically significant difference between the relatively normal and pre-deterioration states. The specific formula for the one-sample t-test statistic is provided below Eq. (S8), and it is used to ascertain whether the constant z significantly deviates from the mean of $\hat{Z} = (z_1, z_2, \cdots, z_n)$.

$$S = \frac{(\text{mean}(\hat{Z}) - z)\sqrt{n}}{\text{SD}(\hat{Z})} \tag{S8}$$

where $\text{mean}(\hat{Z})$ corresponds to the mean of vector $\hat{Z}$, and $\text{SD}(\hat{Z})$ is denoted as its standard deviation. The P-value associated with the index is obtained from the t-distribution to assess the statistical difference between $\text{mean}(\hat{Z})$ and z. A significant statistical difference exists between $\text{mean}(\hat{Z})$ and z if P-value less than or equal to 0.05 ($P < 0.05$). For our research purposes, we consider a time point $t > 2$ as a pre-deterioration state or critical point if the SCNE value H_t satisfies the subsequent two criteria: (i) $H_t > H_{t-1}$ and (ii) H_t displays statistical difference ($P < 0.05$) from the mean of the vector H=$(H_1, H_2, \cdots, H_{t-1})$. When the condition outlined below is met by the SCNE value H_2: (i) H_2 > H_1 and (ii) H_2 is a statistical difference ($P < 0.05$) from the mean of the vector H=(H_1, H_3), the time point $T = 2$ is viewed as the pre-deterioration state.

H. Description of the five real-world datasets

To illustrate the functionality of the SCNE method, it has been applied to five real-world datasets: kidney clear cell carcinoma (KIRC), stomach adenocarcinoma (STAD), and lung adenocarcinoma (LUAD) from the The Cancer Genome Atlas (TCGA) database (http://cancergenome.nih.gov), and influenza infection data (accession number: GSE30550) and single-cell data of epithelial cell deterioration in colorectal cancer (accession number: GSE161277) from the Gene Expression Omnibus (GEO) database (http://www.ncbi.nlm.nih.gov/geo/). The detailed description and sources of the datasets are listed as follows.

The dataset TCGA-KIRC comprises 353 tumor samples and 72 tumor-adjacent samples. By utilizing the clinical information available in TCGA, the tumor samples can be categorized into four stages: stage I (198 samples), stage II (41 samples), stage III (64 samples), and stage IV (50 samples). Access to the gene expression profiling data can be obtained from the following source: https://portal.gdc.cancer.gov/projects/TCGA-KIRC.

The TCGA-STAD dataset consists of 337 tumor samples and 35 tumor-adjacent samples. Based on the corresponding clinical information available in TCGA, the tumor samples are classified into different stages: stage I (50 samples), stage IIA (38 samples), stage IIB (55 samples), stage IIIA (69 samples), stage IIIB (57 samples), stage IIIC (38 samples), and stage IV (30 samples). The gene expression profiling data can be retrieved from the following source: https://portal.gdc.cancer.gov/projects/TCGA-STAD.

The TCGA-LUAD dataset is composed of 500 tumor samples and 59 tumor-adjacent samples. On the basis of the corresponding clinical information of TCGA, tumor samples are divided into stage I (269 samples), stage IIA (50 samples), stage IIB (71 samples), stage IIIA (73 samples), stage IIIB (11 samples), and stage IV (26 samples). Access to the gene expression profiling data can be obtained from the following source: https://portal.gdc.cancer.gov/projects/TCGA-LUAD.

The influenza infection data is generated from gene expression profiles of peripheral blood samples obtained from 17 volunteers who were infected with the H3N2/Wisconsin virus in their nasal cavity. Out of these 17 subjects, severe infection symptoms were observed in nine individuals (subject 1, 5, 6, 7, 8, 10, 12, 13, and 15), while the remaining eight subjects remained healthy. Gene expressions were recorded for these subjects at 16 time points (-24, 0, 5, 12, 21, 29, 36, 45, 53, 60, 69, 77, 84, 93, 101, and 108 hours). For each volunteer, the gene expression profiles of the preceding four time points (-24, 0, 5, and 12 h) were considered as reference samples, while the gene expression at each subsequent time point is considered a case sample. The gene expression profiling data can be downloaded from the Gene Expression Omnibus (GEO) database under accession number GSE30550.

The single-cell data of epithelial cell deterioration in colorectal cancer is processed based on Seurat pipelines. Owing to biological differences between tissues, we removed batch effects from patients using the R package Harmony. The resolution parameter of FindClusters function was set at 1 for all cell types and 0.6 for the epithelial cell subpopulation. By analyzing intracellular gene expression patterns, the progression of epithelial cell deterioration is categorized into six different periods or clusters: cluster 1 (950 cells), cluster 2 (450 cells), cluster 3 (650 cells), cluster 4 (450 cells), cluster 5 (450 cells), and cluster 6 (738 cells). The gene expression profiling data can be obtained from the Gene Expression Omnibus (GEO) database with accession number GSE161277.

References

[1] Foo M, Kim J, Bates DG. Modelling and control of gene regulatory networks for perturbation mitigation. IEEE/ACM Transactions on Computational Biology and Bioinformatics. 2019; 16(2):583-595.

[2] Yan J, Li P, Li Y, Gao R, Bi C, Chen L. Disease prediction by network information gain on a single sample basis. Fundamental Research. 2023.

[3] Koizumi K, Oku M, Hayashi S, Inujima A, Shibahara N, Chen L, Igarashi Y, Tobe K, Saito S, Kadowaki M, Aihara K. Suppression of Dynamical Network Biomarker Signals at the Predisease State (*Mibyou*) before Metabolic Syndrome in Mice by a Traditional Japanese Medicine (Kampo Formula) Bofutsushosan. Evidence-Based

Complementary and Alternative Medicine. 2020, 9129134.
[4] Gao R, Yan J, Li P, Chen L. Detecting the critical states during disease development based on temporal network flow entropy. Briefings in Bioinformatics, 2022, 23(5):bbac164.
[5] Jiayuan Zhong, Huisheng Liu, Pei Chen. Single-sample network module biomarkers (sNMB) reveals the pre-deterioration stage of disease progression. Journal of Molecular Cell Biology, 2022, 14(8), mjac052.
[6] Rui Liu, Jiayuan Zhong, Xiangtian Yu, Yongjun Li, Pei Chen*. Identifying critical state of complex diseases by single-sample-based hidden Markov model, Frontiers in Genetics, 2019, 10: 285.
[7] Rui Liu, Pei Chen, Luonan Chen. Single-sample landscape entropy reveals the imminent phase transition during disease progression. Bioinformatics, 2020, 36(5): 1522-1532.
[8] Gao R, Yan J, Li P, Chen L. Detecting the critical states during disease development based on temporal network flow entropy. Briefings in Bioinformatics. 2022; 23(5):bbac164.
[9] Liang J, Li ZW, Yue CT, Hu Z, Cheng H, Liu ZX, Guo WF. Multi-modal optimization to identify personalized biomarkers for disease prediction of individual patients with cancer. Briefings in Bioinformatics. 2022; 23(5):bbac254.
[10] Liu X, Chang X, Leng S, Tang H, Aihara K, Chen L. Detection for disease tipping points by landscape dynamic network biomarkers. National Science Review. 2019; 6(4):775-785.
[11] Liu X, Chang X, Liu R, Yu X, Chen L, Aihara K. Quantifying critical states of complex diseases using single-sample dynamic network biomarkers. PLoS computational biology, 2017, 13(7):e1005633.
[12] Zhong J, Liu R, Chen P. Identifying critical state of complex diseases by single-sample Kullback-Leibler divergence. BMC Genomics. 2020; 21(1):87.
